# Surveilling Influenza Incidence With Centers for Disease Control and Prevention Web Traffic Data: Demonstration Using a Novel Dataset

**DOI:** 10.2196/14337

**Published:** 2020-07-03

**Authors:** Wendy K Caldwell, Geoffrey Fairchild, Sara Y Del Valle

**Affiliations:** 1 X Computational Physics Division Los Alamos National Laboratory Los Alamos, NM United States; 2 School of Mathematical and Statistical Sciences Arizona State University Tempe, AZ United States; 3 Analytics, Intelligence, and Technology Division Los Alamos National Laboratory Los Alamos, NM United States

**Keywords:** influenza, surveillance, infoveillance, infodemiology, projections and predictions, internet, data sources

## Abstract

**Background:**

Influenza epidemics result in a public health and economic burden worldwide. Traditional surveillance techniques, which rely on doctor visits, provide data with a delay of 1 to 2 weeks. A means of obtaining real-time data and forecasting future outbreaks is desirable to provide more timely responses to influenza epidemics.

**Objective:**

This study aimed to present the first implementation of a novel dataset by demonstrating its ability to supplement traditional disease surveillance at multiple spatial resolutions.

**Methods:**

We used internet traffic data from the Centers for Disease Control and Prevention (CDC) website to determine the potential usability of this data source. We tested the traffic generated by 10 influenza-related pages in 8 states and 9 census divisions within the United States and compared it against clinical surveillance data.

**Results:**

Our results yielded an *r*^2^ value of 0.955 in the most successful case, promising results for some cases, and unsuccessful results for other cases. In the interest of scientific transparency to further the understanding of when internet data streams are an appropriate supplemental data source, we also included negative results (ie, unsuccessful models). Models that focused on a single influenza season were more successful than those that attempted to model multiple influenza seasons. Geographic resolution appeared to play a key role, with national and regional models being more successful, overall, than models at the state level.

**Conclusions:**

These results demonstrate that internet data may be able to complement traditional influenza surveillance in some cases but not in others. Specifically, our results show that the CDC website traffic may inform national- and division-level models but not models for each individual state. In addition, our results show better agreement when the data were broken up by seasons instead of aggregated over several years. We anticipate that this work will lead to more complex nowcasting and forecasting models using this data stream.

## Introduction

### Background and Motivation

Every year, an estimated 5% to 20% of people in the United States become infected with influenza [1]. The typical influenza season begins in October and ends in May, with the peak occurring in the winter months. Annually, 3000 to 50,000 people die from the flu, with another 200,000 people requiring hospitalization [2]. The yearly flu burden is estimated to cost around US $11.2 billion in lost productivity, with some estimates as high as US $87 billion [2,3]. Timely surveillance of influenza can help reduce this burden, allowing health care facilities to more adequately prepare for the influx of patients when flu levels are high [4].

One common surveillance measure is the fraction of patients presenting with influenza-like illness (ILI), consisting of a fever of at least 100°F (37.8°C) and a cough or sore throat with no other known cause [5]. ILI data are collected from about 2900 volunteer health care providers throughout the United States, although each week, only about 1800 of them report their data. These data are then aggregated and made public after a time lag of about 1 to 2 weeks [1,6-10]. As the ILI data are collected from volunteer health care providers, the dataset is incomplete. If policies were enacted to provide incentives for health care providers who report these data or to make reporting compulsory, the result would be a more complete dataset. Other surveillance systems include virological data from the World Health Organization, emergency department visits, electronic health records, crowd-sourced ILI reports, Widely Internet Sourced Distributed Monitoring, Influenzanet, and Flu Near You [11,12].

### Internet Data Streams

In the United States, 87% [13] of adults use the internet. Of those internet users, 72% [13] have used the internet to search for health information within the last year. The most common health-related searches are for information regarding a specific disease or condition (66%) and information about a specific treatment or procedure (56%) [13,14].

There are two main types of health-related internet activity. The first is health sharing, in which internet users post about health-related topics (eg, a tweet about being sick). The second is health seeking, in which users use the internet to obtain information about health-related topics [6]. In this paper, we focused on health-seeking behavior. Previous studies have shown that analyzing web-based health-seeking behavior can improve early detection of disease incidence by detecting changes in disease activity [9,15-19]. Similarly, other studies have shown that internet data emerging from search queries can aid detection of outbreaks in areas with large populations of internet users [20] because web-based health-related search queries and epidemics are often strongly correlated [20,21].

Internet data have been used to forecast disease incidence in other models. Polgreen et al [9] developed linear influenza forecasting models with lags of 1 to 10 weeks for each of the 9 US census regions using search queries from Yahoo [9]. The best performing models had lags of 1 to 3 weeks and an average *r^2^* value of 0.38 (with a high of 0.57 in the East South Central region) [9]. These low *r^2^* values demonstrate potential problems in relying on search information alone. Ginsberg et al [15] were able to predict influenza epidemics 2 weeks in advance using Google search queries to fit linear models using log-odds of ILI visits and related searches.

Using a Poisson distribution and Lasso regression, McIver and Brownstein [8] obtained an *r^2^* value of 0.946 using Wikipedia data, although some data were excluded from analyses because of increased media attention and higher than normal influenza activity. Generous et al [7] used Wikipedia data to train a statistical model with linear regression, which demonstrated its potential for forecasting disease incidence worldwide, including influenza in the United States, which had an *r^2^* value of 0.89. Hickmann et al [1] conducted a similar study of linear regression models, which showed that using Wikipedia to forecast influenza in the United States for the 2013 to 2014 season resulted in an *r^2^* value greater than 0.9 in some instances.

Integrating both Wikipedia data and Google Flu Trends, Bardak et al [22] obtained *r^2^* values of 0.94 and 0.91 using ordinary least squares (OLS) and ridge regression, respectively, for forecasting influenza outbreaks. For OLS nowcasting, the *r^2^* value was 0.98 in the best case. For the best fit, the weekly data were offset by 1 week, so that ILI visits were correlated with internet data from the prior week [22].

As part of the Centers for Disease Control and Prevention (CDC)’s 2013 to 2014 Predict the Influenza Season Challenge, 9 teams used digital data sources to create forecasting models. The digital sources these teams used were Wikipedia, Twitter, Google Flu Trends, and HealthMap. The teams used either mechanistic or statistical models to create their forecasts, with the most successful team using multiple data sources, which may have reduced biases usually associated with internet data streams [23]. Broniatowski et al [24] used Twitter data to detect increasing and decreasing influenza prevalence with 85% accuracy. Zhang et al [25] used Twitter data to inform stochastically, spatially structured mechanistic models of influenza in the United States, Italy, and Spain.

Internet data streams have also been used to supplement traditional surveillance techniques with nowcasting models. Paul et al [26] used Twitter along with ILI data from the CDC to produce nowcasting influenza models as well as nowcasting models using solely ILI data. They concluded that the addition of Twitter data led to more accurate nowcasting models. Santillana et al [27] combined Google Trends data and CDC-reported ILI data to create models for nowcasting and forecasting influenza. Lampos et al [28] used search query data to explore both linear and nonlinear nowcasting models. Yang et al [29] used Google search data to create an influenza tracking model with autoregression.

In contrast, we considered data on page views of the CDC website rather than search data from sites not solely devoted to public health. We used this dataset because we expect it to be inherently less noisy because of its focus on public health issues. We used OLS to nowcast influenza nationally, across the 9 US census divisions, and across 8 states using access data from 10 influenza-related CDC pages. Our nowcasting models cover influenza seasons from 2013 to 2016, with the 2012 to 2013 season being partially included because the CDC page view dataset begins on January 1, 2013. The inclusion of an incomplete influenza season serves to inform if this dataset can be used given a more restrictive time frame. We included both positive and negative results to advance our knowledge regarding when internet data may or may not work. The negative results are crucial to advancing the field of disease surveillance using internet data, as they demonstrate when these data sources contribute to unreliable surveillance. We focus on answering the following two research questions: (1) Can CDC page visits be used as an additional data source for monitoring disease incidence? and (2) What is the appropriate time shift of the page view data needed to obtain the best data fit?

## Methods

### Data Sources

We used page view data provided by the CDC. Each data point contains the page name, date and time of access, and the geographic location from where the page was viewed. These data are available at geographic resolutions of national and state levels and include some metropolitan areas (eg, New York City). The data are available at a number of temporal resolutions beginning on January 1, 2013. For these models, we used weekly page view data to coincide with the ILI data temporal resolution. The data are available as raw page view counts and page view counts normalized with respect to all CDC page views, and we considered the latter for this work. We selected pages associated with general influenza information, treatment, and diagnosis. Pages were sometimes renamed, but we were able to follow the evolution of each selected page by using keywords in the page titles as well as the date ranges for available data.

As the majority of health-related internet searches concern specific conditions, treatments, and procedures [14], we selected pages related to those topics. These pages also align with the study by Johnson et al [30], who used pages in the categories of diagnosis and treatment as well as prevention and vaccination for influenza surveillance [30]. Specifically, we used the following pages: antivirals, flu basics, FluView, high risk complications, key facts, prevention, symptoms, treating influenza, treatment, and vaccine. We then aggregated the page views of interest for each of our models. FluView has the potential to be an outlier page, especially when used alone, as this page tracks the severity of the influenza season and could have higher page views as a result of media attention and severe influenza seasons. However, when combined with other pages focused on treatment and prevention, we expected these page view data to be useful for our models. A complete list of pages can be found in Multimedia Appendix 1.

The states we selected were based on the severity of flu (determined from FluView) during the available seasons and the availability of ILI data at the time of the study, which is not standardized and is dependent on each state’s reporting mechanism. ILI data for each state include the week ending or starting date and the percentage of ILI for the specified week. Although some states also report additional data, such as school closures and hospitalizations, these data are not made available by every state. Note that ILI reporting and accessibility vary across all states. The states we selected were (1) California, (2) Maine, (3) Missouri, (4) New Jersey, (5) New Mexico, (6) North Carolina, (7) Texas, and (8) Wisconsin. With the exception of Texas, these states did not release ILI data outside of the typical flu season. As the purpose of this study was to demonstrate the viability of nowcasting, we considered only those ILI data available during the study period. Although some states have made their ILI more accessible since the end of the study, we did not consider these data, as they were not available during the study period. The exclusion of additional data not available during the study period helps to preserve the premise of nowcasting by focusing only on data sources available during the study period. Likewise, our state ILI data often came from the state’s individual weekly reports during the seasons used in the study. A complete list of the data sources for the state ILI can be found in Multimedia Appendix 2, and the clinical data are available in Multimedia Appendices 3 and 4.

**Figure 1:**
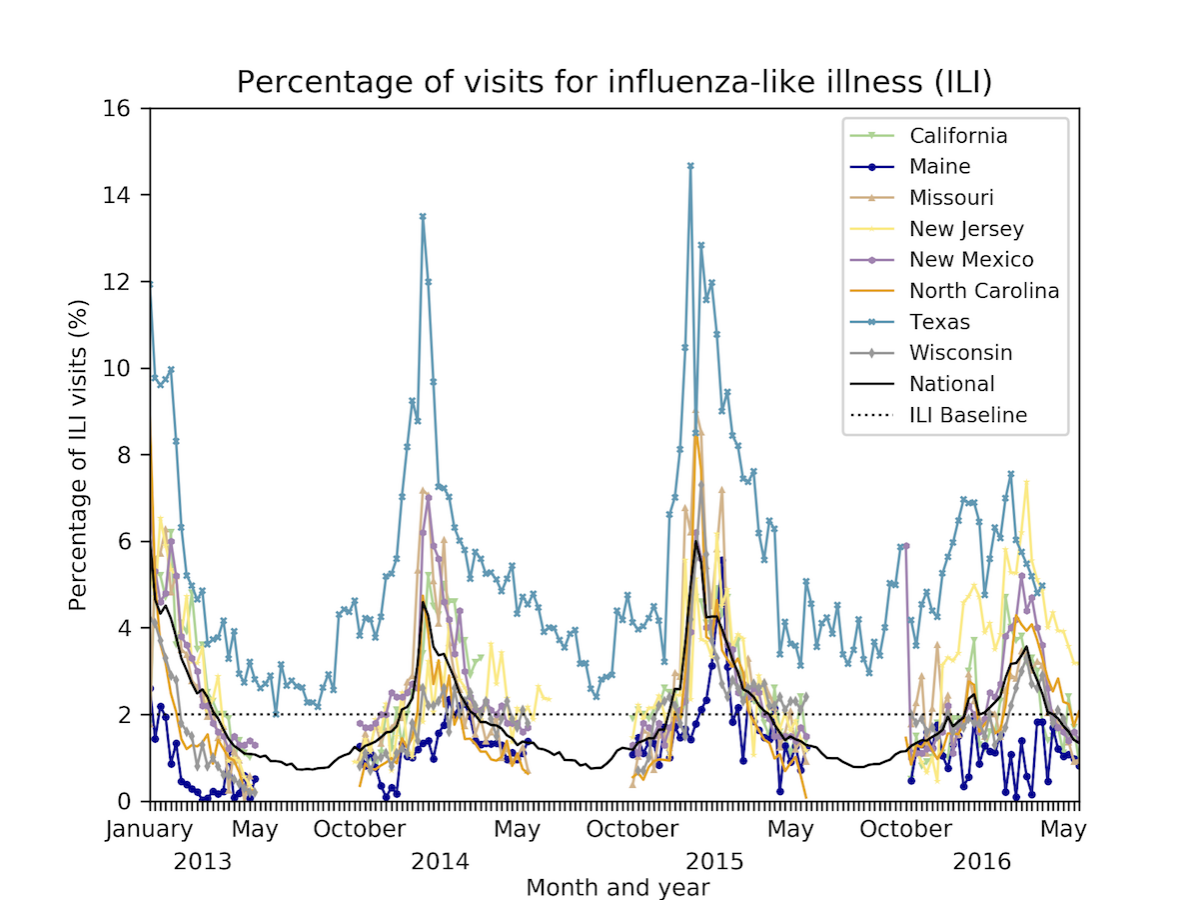
Percentage of ILI visits per state compared with the typical national baseline of 2%. Maine (dark blue) and Texas (teal) exhibit outlier behavior, with Texas having a greater ILI percentage and Maine having a lesser ILI percentage. The remaining states follow the national ILI trend, shown in black. ILI: influenza-like illness.

**Figure 2:**
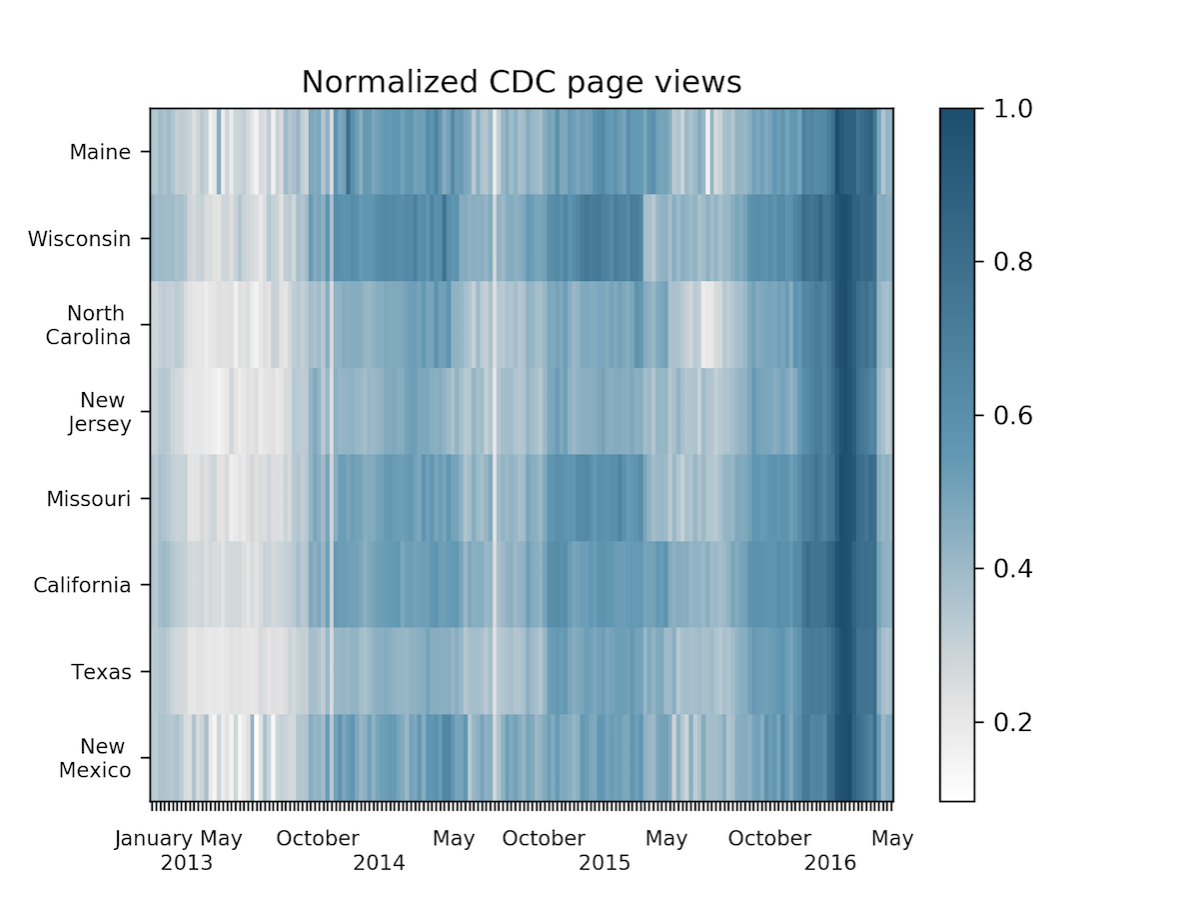
Normalized CDC web traffic as a heat map. Darker areas indicate more page views and appear to correlate with increases in influenza-like illness. The page views also appear to be more prevalent during the typical influenza season, October to May. CDC: Centers for Disease Control and Prevention.

Figure 1 shows the percentage of ILI visits for each state considered in this study and the national percentage of ILI visits. We see distinct spikes that indicate the peaks of the flu seasons. With the exception of Maine, which behaves as an outlier at times, the figure shows spikes indicating there are peak weeks for influenza-related page views. Texas also exhibits outlier behavior with ILI percentages consistently higher than the typical national baseline of 2%, which is used to determine when the flu has reached epidemic status. These 2 outliers are shown in teal (Texas) and dark blue (Maine). The national ILI is shown in black. The remaining states exhibit behavior consistent with the national ILI trend. Figure 2 shows the CDC page view data as a heat map: weeks with more page views are shown darker than weeks with fewer page views.

In addition to selected states, we also considered the 9 US census divisions: New England, Middle Atlantic, East North Central, West North Central, South Atlantic, East South Central, West South Central, Mountain, and Pacific. Multimedia Appendix 5 provides a list of states included in each division. Data for the census divisions were obtained from the CDC and are presented in Multimedia Appendix 3.

### Linear Regression

**Figure 3:**
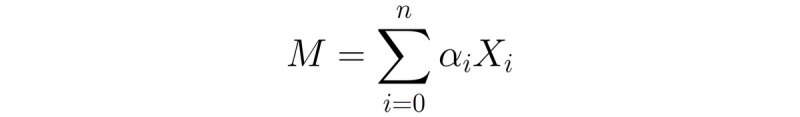
Mathematical formula of the linear ILI models created in this study. The model M represents the fraction of ILI visits, where <inline-graphic xlink:href="jmir_v22i6e14337_fig7.png" mimetype="image" xlink:type="simple"/> are the regression coefficients and *X*=1, *X*_1_, ... *X*_n_ is the vector of CDC page view data, with n representing the number of CDC pages used for the model, ranging from 1 to 10. ILI: influenza-like illness; CDC: Centers for Disease Control and Prevention.

We used statsmodels version 0.9.0 [31], a statistical analysis module for Python, to perform linear regression on our datasets using OLS. This creates a linear model *M*, the summation of regression coefficients multiplied by page view data. Figure 3 shows the mathematical formula of *M*, where 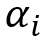 are the regression coefficients and *X*=1, *X*_1_, ... *X*_n_ is the vector of CDC page view data, with *n* representing the number of CDC pages used for the model, ranging from 1 to 10. *M* is a value between 0 and 1, representing the fraction of ILI visits. To plot the models and data on the same axes, we normalized *M* for visualization purposes, with *M=1* corresponding to the ILI percentage during the peak week of the influenza season. We correlated ILI and CDC page views for the same week or with a 1-week shift. In the shifted cases, we shifted the ILI data forward by 1 week, so that the model associates the current week’s page views with the following week’s ILI data. This shift is performed to account for the incubation period of influenza and the time between the onset of symptoms and the first doctor visit. Statsmodels [31] uses the CDC page view and ILI data to determine the appropriate regression coefficients; fits parameters with OLS; and computes the goodness-of-fit, *r*^2^, also referred to as the coefficient of determination. The *r*^2^ value measures how well 2 time series correlate. An *r*^2^ value of 1 indicates a perfect fit, whereas an *r*^2^ value of 0 indicates no correlation. Although *r*^2^ is not necessarily the best metric to judge goodness-of-fit [6], it is nonetheless the most common metric used and still provides one with a decent overall sense of fit quality. In addition, we examined the root mean square error (RMSE) and the normalized root mean square error (NRMSE) using Python scikit-learn libraries. The RMSE and NRMSE metrics measure how the model prediction differs from the actual data, with the NRMSE normalized so that the greatest possible value is 1. For these metrics, lower numbers indicate a better fit.

## Results

### Format of Results

We analyzed the data at the national, division, and state levels and computed *r*^2^ for each geographic resolution. In this section, we discuss the results of our experiments, both successes and failures. We include figures of models at the national, census division, and state levels. Owing to the varying scales between page views and ILI percent, we chose to normalize the data and our models to plot them on the same axes. Raw data were used to create the models and then each model was normalized with respect to its maximum. We also normalized the ILI data and CDC web traffic data with respect to their maximums for the given period so that all 3 curves may appear in the same plot. Additional model successes and failures not discussed here can be found in Multimedia Appendix 6.

### National Results

**Figure 4:**
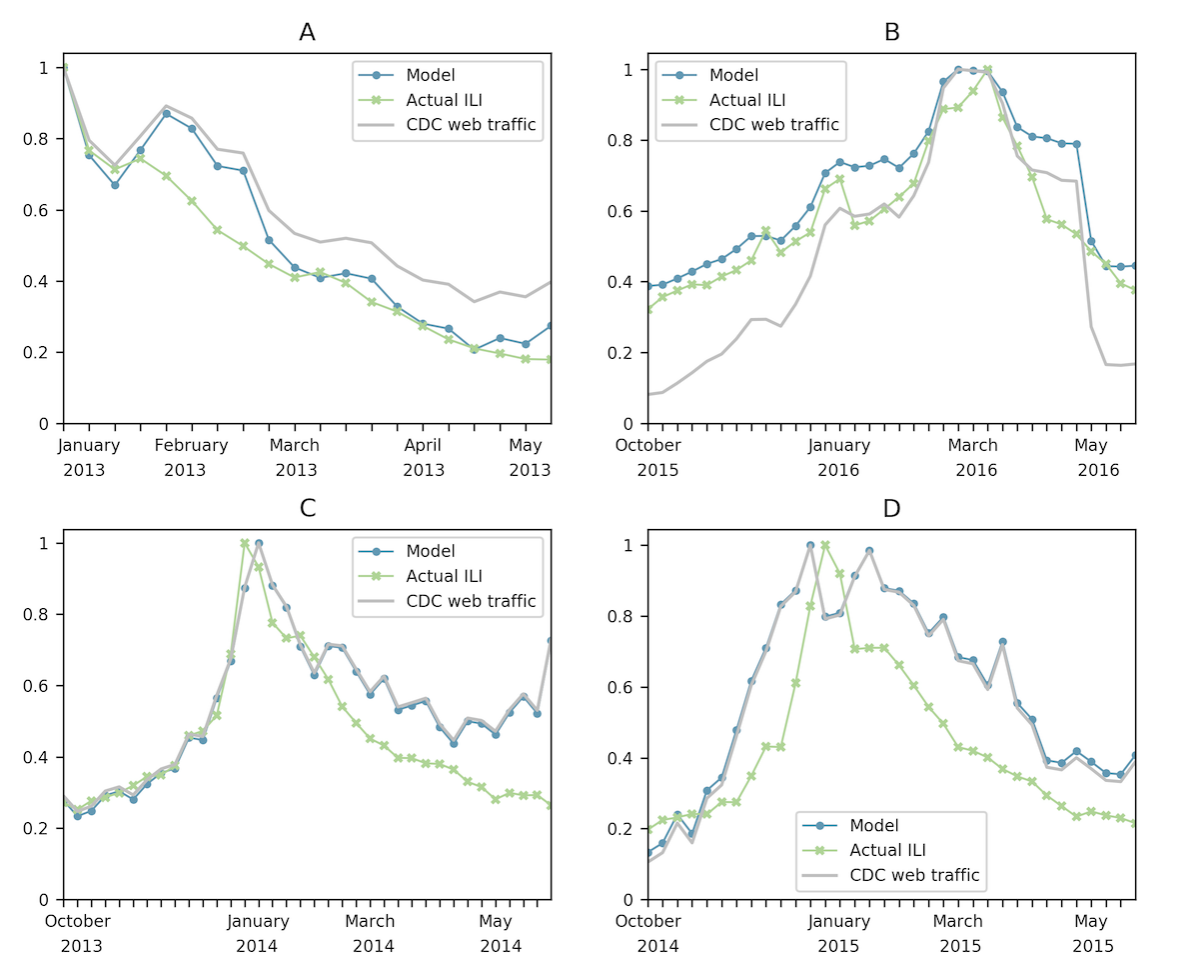
These plots show national models and the associated pages and influenza seasons. (A) FluView, Symptoms, and Treatment, 2012 to 2013. (B) Symptoms, 2015 to 2016. (C) FluView, 2013 to 2014. (D) Antivirals and Prevention, 2014 to 2015. CDC: Centers for Disease Control and Prevention; ILI: influenza-like illness.

We selected pages that corresponded to the topics most often searched during web-based health-seeking activities. Aggregating all 10 pages in a single model, we were able to achieve an *r^2^* value of 0.889 for the national 2012 to 2013 influenza season after implementing a 1-week shift. We also succeeded in modeling the national 2015 to 2016 influenza season with no shift, achieving an *r^2^* value of 0.834. We obtained better results when limiting the pages to FluView, Symptoms, and Treatment, which we attribute to the information on these pages aligning with topics most commonly used for internet health seeking. For these pages, the most successful models did not have a shift. For the 2012 to 2013 influenza season, we achieved an *r^2^* value of 0.906. The model for the 2015 to 2016 season had an *r^2^* value of 0.891. Table 1 shows the most successful model for each influenza season included in this study. Figure 4 shows these models, with each figure caption indicating which page(s) comprise CDC web traffic, which appears in each figure and are the data used in the model.

**Table 1:** Pages and shifts for the most successful models for each influenza season at the national level.

Pages used in model	Season	Shift	*r^2^*	Root mean square error	Normalized root mean square error
FluView, Symptoms, and Treatment	2012-2013	None	0.912	0.423	0.070
Symptoms	2015-2016	None	0.892	0.213	0.060
FluView	2013-2014	None	0.802	0.510	0.111
Antivirals and Prevention	2014-2015	None	0.778	0.615	0.103

### Census Division Results

**Figure 5:**
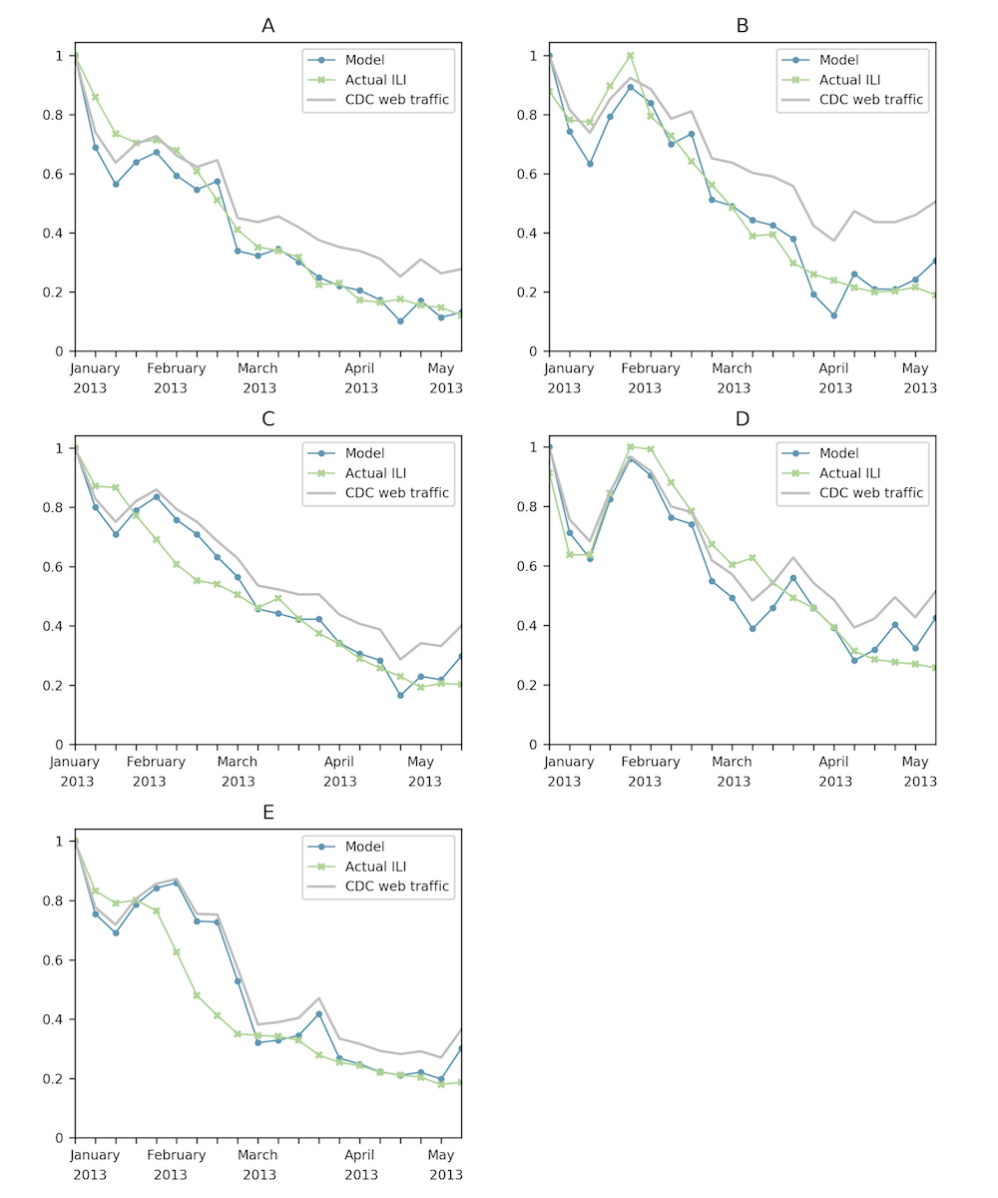
Census division model successes using the FluView, Symptoms, and Treatment pages for the 2012 to 2013 influenza season. (A) West North Central, 2012 to 2013. (B) Mountain, 2012 to 2013. (C) East North Central, 1-week shift, 2012 to 2013. (D) Pacific, 2012 to 2013. (E) West South Central 2012 to 2013. These plots represent the census division models that had the highest *r^2^* value in the 2012 to 2013 influenza season. CDC: Centers for Disease Control and Prevention; ILI: influenza-like illness.

Using the data for each of the 9 census divisions, we were able to achieve an *r^2^* value greater than 0.7 for at least one case for each division. We considered all seasons together and separately, with the better results obtained from modeling each individual season. We considered all 10 pages together as well as combinations of one or more of these pages. In the most successful case, the model was able to closely match the 2015 to 2016 influenza season for the West North Central division, with an *r^2^* value of 0.955 using the FluView, Symptoms, and Treatment pages. Although we had successes using all 10 pages, the most successful model for each division involved only these 3 pages. Figure 5 shows some of these models, and Table 2 highlights these successes.

**Table 2:** The 9 census divisions and the season and shift for which the division’s model had the highest *r^2^* value. The table also shows the root mean square error and the normalized root mean square error. The results presented correspond to the FluView, Symptoms, and Treatment pages aggregated.

Division	Season	Shift	*r* ^2^	Root mean square error	Normalized root mean square error
West North Central	2012-2013	None	0.955	0.367	0.057
Mountain	2012-2013	None	0.921	0.336	0.077
New England	2015-2016	None	0.920	0.096	0.096
East North Central	2012-2013	1 week	0.899	0.331	0.076
South Atlantic	2015-2016	None	0.893	0.218	0.065
Middle Atlantic	2015-2016	None	0.861	0.302	0.073
Pacific	2012-2013	None	0.849	0.503	0.094
West South Central	2012-2013	None	0.828	0.986	0.105
East South Central	2015-2016	1 week	0.793	0.365	0.082

### State Results

We found *r^2^* for each of the states considered in this study, using a variety of pages and page combinations. Table 3 lists the most successful models for each state, the season, the data shift, and the *r^2^* value.

**Table 3:** The most successful results for each state considered in this study.

State	Page(s)	Season	Shift	*r* ^2^	Root mean square error	Normalized root mean square error
Texas	All^a^	2012-2013	1 week	0.930	0.667	0.067
Wisconsin	FVST^b^	2012-2013	None	0.833	0.533	0.127
New Jersey	All	2012-2013	1 week	0.832	0.767	0.117
Missouri	FVST	2012-2013	1 week	0.823	0.801	0.127
North Carolina	FVST	2015-2016	1 week	0.781	0.455	0.106
New Mexico	All	2015-2016	1 week	0.771	1.184	0.197
California	FVST	2012-2013	1 week	0.758	0.777	0.125
Maine	Antivirals	2012-2013	None	0.662	0.445	0.171

^a^*All* refers to the aggregation of all 10 pages.

^b^*FVST* refers to the aggregation of the FluView, Symptoms, and Treatment pages.

**Figure 6:**
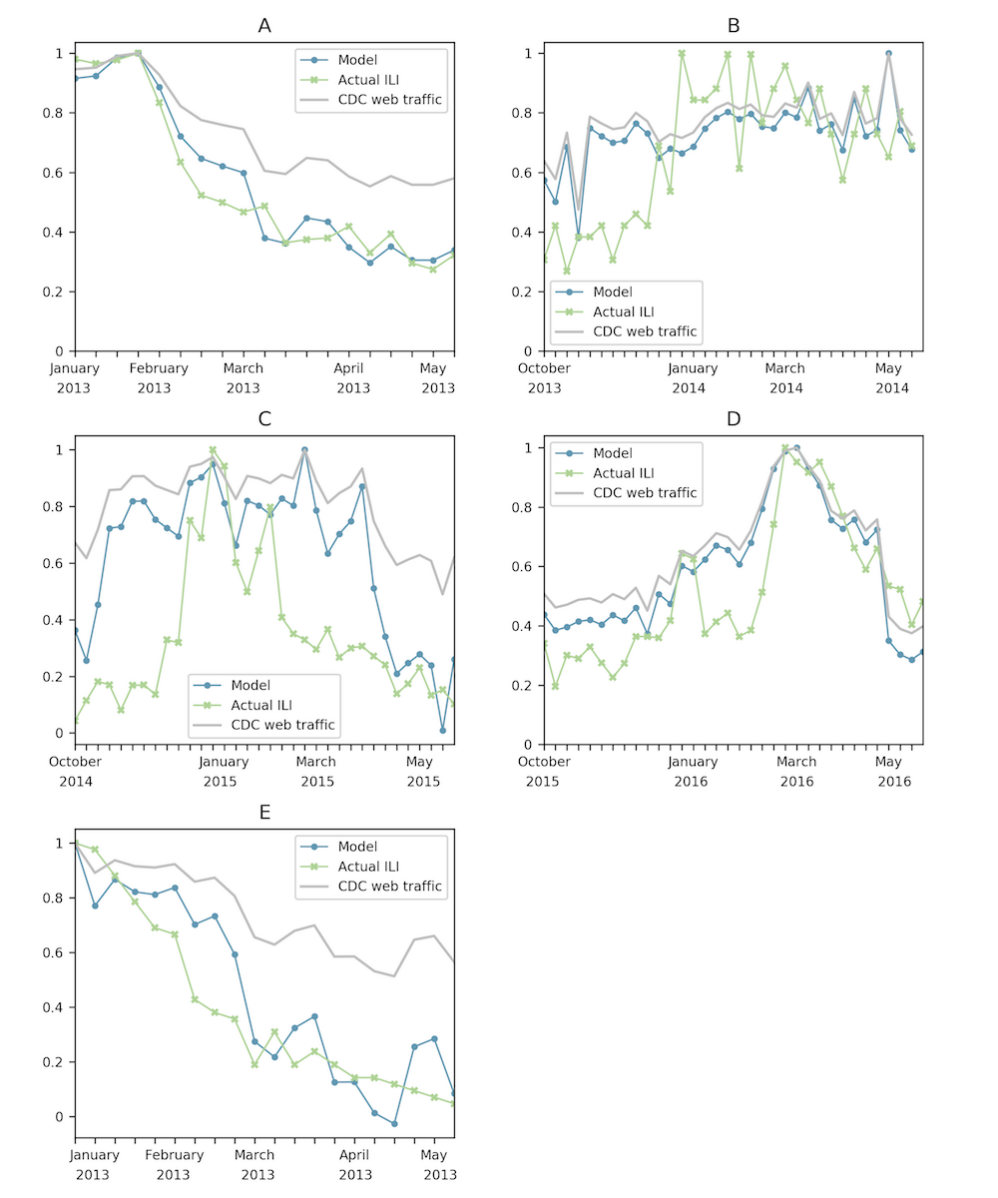
Different states during different seasons. (A) Texas, 1-week shift, 2012 to 2013. (B) Wisconsin, 2013 to 2014. (C) Missouri, 2014 to 2015. (D) North Carolina, 2015 to 2016. (E) Wisconsin 2012 to 2013. The *r^2^* values of each of these models ranged from 0.187 to 0.930. These models aggregated all 10 pages, and the success varied by state. CDC: Centers for Disease Control and Prevention; ILI: influenza-like illness.

Figure 6 shows both successes and failures at the state level. Adding all the pages together, we were able to obtain *r^2^* values of 0.930 and 0.801 for Texas and Wisconsin, respectively, for the 2012 to 2013 influenza season. For the 2013 to 2014 season, the highest *r^2^* value was 0.187 for Wisconsin. For the 2014 to 2015 season, the highest *r^2^*value was 0.322 for Missouri. For the 2015 to 2016 season, the highest *r^2^* value was 0.647 for North Carolina.

We were not surprised that Texas had the best fit. Texas was the only state we included that provided ILI data not only for the typical influenza season but also for the off-season. These additional data likely contributed to the success of the Texas models. In keeping with our nowcasting scenario, we only included data available during the study period. During that period, Texas was the only state that provided off-season ILI data. These data have since been made available from other states, but the availability was not present during the study. The lack of success we encountered in modeling Maine was also expected because of Maine’s outlier behavior in ILI, having values considerably lower and out of pattern with other states. The models in Figure 6 included all 10 pages aggregated together. However, as indicated by the individual state results, this does not always lead to the best fit. Successful models often include a combination of select pages (such as FluView, Symptoms, and Treatment) but not an aggregation of all 10. Furthermore, aside from Texas, we did not have ILI data for the states outside of the typical flu season. Without these additional data, we are unable to determine how strongly the lower page views in the off-season correlate with off-season ILI.

We then shifted the ILI data forward by 1 week. The regression analysis yielded 7 state/season combinations with *r^2^* values greater than 0.7 (Table 4). The table also includes both the regular RMSE and NRMSE.

**Table 4:** States with models that had an *r^2^* value greater than 0.7 when aggregating all 10 pages and shifting the influenza-like illness data forward by 1 week. The regular and normalized root mean square errors are also displayed.

State	Season	*r* ^2^	Root mean square error	Normalized root mean square error
Texas	2012-2013	0.930	0.667	0.067
New Jersey	2012-2013	0.832	0.767	0.117
New Mexico	2015-2016	0.771	1.184	0.197
California	2012-2013	0.746	0.797	0.129
Wisconsin	2012-2013	0.727	0.626	0.153
North Carolina	2015-2016	0.708	1.028	0.204
Missouri	2012-2013	0.702	1.039	0.165

Adding only the FluView, Symptoms, and Treatment pages, we obtained an *r*^2^ value of 0.7 or greater for 6 state/season combinations. For the 2013 to 2014 season, the highest *r*^2^ values were 0.612 for California and 0.568 for Wisconsin. Although this is still less than desired, it is a vast improvement from the *r*^2^ values found from adding all 10 pages. For the 2014 to 2015 season, the highest *r*^2^ was 0.575 for Missouri. Again, although the correlation appears to be weak, it is a stronger correlation than taking all 10 pages together. Using the same 3 pages and implementing a 1-week shift, we obtained an *r*^2^ value of 7 or greater for 10 state/season combinations. For the 2014 to 2015 season, the highest *r*^2^ value was 0.548 for Missouri.

### State Influenza-Like Illness Data Availability

The purpose of this study was to demonstrate the viability of near real-time nowcasting during the influenza seasons from 2013 to 2016. To maintain the premise of nowcasting, we chose states with publicly available data, or data available on request, during the period of the study. During the study period, state ILI data were not readily available on the CDC website. Instead, we had to rely on data available through state health-related organizations for each state. In addition, throughout the course of influenza seasons, ILI numbers are often updated as delayed data are reported and made available. However, because we are focusing our study on a nowcasting scenario, we do not consider the ILI data from those seasons as they are reported today but rather as they were reported during the study period.

### Model Failures

We generally found the models to be successful when considering pages most closely related to typical health-seeking behavior and when considering each flu season individually. When trying to model multiple influenza seasons together, we had a number of unsuccessful models. Considering all pages and national ILI data, the model combining the 2012 to 2013 and 2013 to 2014 influenza seasons had an *r*^2^ value of 0.061 and RMSE of 0.553. The combined 2013 to 2014 and 2014 to 2015 model had an *r*^2^ value of 0.241 and RMSE of 0.208. The combined 2014 to 2015 and 2015 to 2016 model had an *r*^2^ value of 0.251 and RMSE of 0.286. At the state level, combining all pages resulted in a number of unsuccessful models. For the 2013 to 2014 season, the Wisconsin model had an *r*^2^ value of 0.187 and RMSE of 0.523. For the 2014 to 2015 season, the Missouri model had an *r*^2^ value of 0.322 and RMSE of 1.845. Model failures not included in this section can be found in Multimedia Appendix 6.

We speculate that a number of factors could contribute to these negative results. Although influenza is a seasonal disease, similar strains can span multiple years, affecting the susceptible populations in subsequent years. Our data stream may be biased toward individuals with more awareness of the CDC. Furthermore, individuals who search for influenza information in one season may not search for that information the next year. Finally, with the exception of Texas, we only have ILI data for the influenza season itself. Thus, although we do have internet data for off-season influenza page views, we do not have corresponding ILI data.

## Discussion

### Conclusions

Internet surveillance data have proven beneficial in predicting ILI incidence during flu seasons. However, our results show that the benefit of internet data streams on informing disease is inconclusive; that is, this study shows that the CDC website traffic can be informative in some cases (eg, national level) but not in others (eg, state level). To determine the extent, we must return to our original research questions.

#### Research Question 1

Given the successes of some of our models, we can conclude that CDC page view data can be used as an additional data source for monitoring disease incidence in some cases (eg, at the national level). The degree to which these data can be used appears to rely on the page selection and time frame. The results of the best models varied across geographic and temporal resolutions, but some trends were consistent in most cases. We obtained successful nowcasts when selecting pages related to topics most commonly used for web-based health queries (specific diseases and treatments) during the time span of a typical influenza season. Longer time spans and pages less associated with specific diseases and treatments led to less successful models. Outlier behavior, such as the ILI data in Maine, affected our models and resulted in less successful models than states with ILI curves exhibiting expected behavior. These results can assist others in selecting appropriate supplemental datasets for disease surveillance as well as appropriate spatial and temporal resolutions.

#### Research Question 2

We obtained our most successful results using a 1-week shift. Moreover, 2-week shifts were successful in some cases but were overall less correlated than 1-week shifts (Multimedia Appendix 6). Using no shift at all proved successful in some cases but not in others. We surmise that the shift required for the best fit depends on the incubation period for the disease in question as well as the period of reporting. The CDC internet data are available daily; however, ILI data are available weekly, so we are limited in the types of shifts we can apply to the datasets. Another factor that could contribute to the need for a 1-week shift is the amount of time between the page view and the subsequent visit to a health care center. If there are one or more days between the page view and the visit, then these 2 events could occur during different weeks. Shifting the data by 1 week accounts for this behavior.

### Future Work

We conclude that more studies on internet data streams are needed to understand when and why internet data work. Our methods are consistent with other feasibility studies and provide insight into the conditions under which internet data streams may inform influenza models. Future work should include rigorously testing the predictive power of the models by separating data into training and testing sets [6].

More studies on geographic resolution could provide a better insight into why some models outperform others at various spatial resolutions. National models across single influenza seasons performed well, with each season included in the study having at least one model with an *r*^2^ value greater than 0.75. We attribute the national model successes to the representation of all 50 states. Internet access may not be as prevalent in all states, but the inclusion of all 50 states allows for more data to be considered. Likewise, the census division models performed well, with overall *r*^2^ values greater than those achieved from national models. Each census division had at least one model in the study with an *r*^2^ value greater than 0.79. We attribute these successes to not only the inclusion of all states but also the division into geographic areas. There are instances in which a person may live in one state and seek medical care in another, perhaps because of working in a neighboring state. These instances are not accounted for by simply looking at states but can be accounted for by considering several neighboring states for 1 model. At the state level, models were overall less successful than at national and census division levels, but each state considered in the study had at least one model with an *r*^2^ value greater than 0.65, and all but Maine had models with an *r*^2^ value greater than 0.75. We attribute the overall lower success of state models to a combination of varying levels of internet access across populated and rural areas, the possibility of people living near neighboring states seeking health care in another state, and the inconsistencies in data availability during the study period. As our study focused on using data sources available during the study, we were limited in the states we could model because of the scarcity of the data.

More studies on temporal resolution could provide a better insight into how best to model seasonal diseases over multiple seasons. Models across multiple seasons were not successful, which we attribute in part to the off-season ILI data being unavailable during the study period. As influenza is a seasonal disease, modeling multiple seasons with 1 model may not be the correct approach, and our multiseason models support this idea. However, more exhaustive studies are needed to draw definitive conclusions on the appropriate spatial resolution for modeling influenza.

## Appendix

Multimedia Appendix 1 Names and date ranges of web pages used in this study.

Multimedia Appendix 2 Sources for state influenza-like illness data used in this study.

Multimedia Appendix 3 Influenza-like illness data for the nine census divisions.

Multimedia Appendix 4 State influenza-like illness data.

Multimedia Appendix 5 The nine US census divisions, listing all states in each division.

Multimedia Appendix 6 Comprehensive list of all models in this study not included in the main text. The list includes model successes and model failures.

## Abbreviations

CDC: Centers for Disease Control and Prevention
ILI: influenza-like illness
NRMSE: normalized root mean square error
OLS: ordinary least squares
RMSE: root mean square error
