# Supplementary material for: Surveilling Influenza Incidence With Centers for Disease Control and Prevention Web Traffic Data: Demonstration Using a Novel Dataset
Source: J Med Internet Res. 2020 Jul 3;22(7):e14337. doi: 10.2196/14337 (PMC7367534; doi:10.2196/14337)
Supplement: Multimedia Appendix 1 [file jmir_v22i7e14337_app1.docx]

## Appendix A: CDC Pages

This section contains the names and time frames for each page used. Some of the pages were renamed during the time period covered here.

## Antivirals

| **Dates** | **Page Name** |
| --- | --- |
| 1/1/13–10/20/13 | CDC–Seasonal Influenza (Flu)–Antivirals |
| 10/20/13–8/10/14 | CDC–Antiviral Dosage \| Health Professionals \| Seasonal Influenza (Flu) |
| 8/24/14–5/28/16 | Antiviral Dosage \| Health Professionals \| Seasonal Influenza (Flu) |

## Flu Basics

| **Dates** | **Page Name** |
| --- | --- |
| 1/1/13–10/13/13 | CDC–Seasonal Influenza (Flu)–Seasonal Influenza Q & A: Flu Basics |
| 10/13/13–6/8/14 | CDC–Seasonal Influenza: Flu Basics \| Seasonal Influenza (Flu) |
| 6/8/14–7/27/14 | Seasonal Influenza: Flu Basics \| Seasonal Influenza (Flu) |
| 7/27/14–8/31/14 | Seasonal Influenza: Flu Basics \| About (Flu) \| CDC |
| 8/31/14–5/28/16 | Seasonal Influenza: Flu Basics \| Seasonal Influenza (Flu) \| CDC |

## FluView

| **Dates** | **Page Name** |
| --- | --- |
| 1/1/13–10/26/14 | CDC–Seasonal Influenza (Flu)–FluView Interactive |
| 10/26/14–5/28/16 | FluView Interactive \| Seasonal Influenza (Flu) \| CDC |

## High Risk Complications

| **Dates** | **Page Name** |
| --- | --- |
| 1/1/13–10/13/13 | CDC–Seasonal Influenza (Flu)–People at High Risk of Developing Flu-Related Complications |
| 10/13/13–6/8/14 | CDC–People at High Risk of Developing Flu-Related Complications \| Seasonal Influenza (Flu) |
| 6/8/14–7/27/14 | CDC–People at High Risk of Developing Flu-Related Complications \| Seasonal Influenza (Flu) |
| 8/17/14–5/28/16 | CDC–People at High Risk of Developing Flu-Related Complications \| Seasonal Influenza (Flu) \| CDC |

## Key Facts

| **Dates** | **Page Name** |
| --- | --- |
| 1/1/13–10/27/13 | CDC–Seasonal Influenza (Flu)–Key Facts About Seasonal Flu Vaccine |
| 10/27/13–7/13/14 | CDC–Key Facts About Seasonal Flu Vaccine \| Seasonal Influenza (Flu) |
| 7/13/14–7/27/14 | Key Facts About Seasonal Flu Vaccine \| Seasonal Influenza (Flu) |
| 7/27/14–5/28/16 | Key Facts About Seasonal Flu Vaccine \| Seasonal Influenza (Flu) \| CDC |

## Prevention

| **Dates** | **Page Name** |
| --- | --- |
| 1/1/13–10/20/13 | CDC–Seasonal Influenza (Flu)–Prevention Strategies for Seasonal Influenza in Healthcare Settings |
| 10/27/13–6/1/14 | CDC–Prevention Strategies for Seasonal Influenza in Healthcare Settings \| Healthcare Professionals \| S… |
| 6/8/14–5/28/16 | Prevention Strategies for Seasonal Influenza in Healthcare Settings \| Health Professionals \| Seaona… |

## Symptoms

| **Dates** | **Page Name** |
| --- | --- |
| 1/1/13–10/13/13 | CDC–Seasonal Influenza (Flu)–Flu Symptoms & Severity |
| 10/20/13–6/8/14 | CDC–Flu Symptoms & Severity \| Seasonal Influenza (Flu) |
| 6/15/14–7/27/14 | Flu Symptoms & Severity \| Seasonal Influenza (Flu) |
| 7/27/14–8/31/14 | Flu Symptoms & Severity \| About (Flu) \| CDC |
| 9/7/14–5/28/16 | Flu Symptoms & Severity \| Seasonal Influenza (Flu) \| CDC |

## Treating Influenza

| **Dates** | **Page Name** |
| --- | --- |
| 1/1/13–2/28/16 | CDC–Seasonal Influenza (Flu)–Q & A: Treating the Flu |

## Treatment

| **Dates** | **Page Name** |
| --- | --- |
| 1/1/13–10/13/13 | CDC–Seasonal Influenza (Flu)–H3N2v Treatment |
| 10/13/13–6/8/14 | CDC–Treatment–Antiviral Drugs \| Seasonal Influenza (Flu) |
| 6/8/14–7/27/14 | Treatment–Antiviral Drugs \| Seasonal Influenza (Flu) |
| 7/27/14–5/28/16 | Treatment–Antiviral Drugs \| Seasonal Influenza (Flu) \| CDC |

## Vaccine

| **Dates** | **Page Name** |
| --- | --- |
| 1/1/13–9/7/14 | CDC–Seasonal Influenza (Flu)–Vaccination |
| 8/31/14–5/28/16 | Cell-based Flu Vaccines \| Seasonal Influenza (Flu) \| CDC |
