# Supplementary material for: Surveilling Influenza Incidence With Centers for Disease Control and Prevention Web Traffic Data: Demonstration Using a Novel Dataset
Source: J Med Internet Res. 2020 Jul 3;22(7):e14337. doi: 10.2196/14337 (PMC7367534; doi:10.2196/14337)
Supplement: Multimedia Appendix 2 [file jmir_v22i7e14337_app2.docx]

## Appendix B: State ILI Sources

This section contains the data sources for the state ILI percentages. Internet sources were accessed May – June 2016.

## California

ILI data were obtained from the California Department of Public Health website at the following URL:

- http://www.cdph.ca.gov/data/statistics/Pages/CISPDataArchive.aspx

## Maine

ILI data were obtained from the State of Maine Department of Health and Human Services website at the following URL:

- http://www.maine.gov/dhhs/mecdc/infectious-disease/epi/influenza/influenza-surveillance-archives.htm

## Missouri

ILI data were obtained from the Missouri Department of Health & Senior Services website at the following URL:

- http://www.health.mo.gov/living/healthcondiseases/communicable/influenza/reports.php

## New Jersey

ILI data were obtained from the State of New Jersey Department of Health website at the following URLs:

- http://www.state.nj.us/health/flu/archives/shtml
- http://www.state.nj.us/health/flu/fluinfo.shtml

## New Mexico

ILI data were obtained from the New Mexico Department of Health website at the following URL:

- https://nmhealth.org/about/erd/ideb/isp/data/

## North Carolina

ILI data were obtained from the North Carolina Health and Human Services website at the following URLS:

- http://epi.publichealth.nc.gov/cd/flu/figures/flu1213.pdf
- http://epi.publichealth.nc.gov/cd/flu/figures/flu1314.pdf
- http://epi.publichealth.nc.gov/cd/flu/figures/flu1415.pdf
- http://flu.nc.gov/data/documents/flu1516.pdf

## Texas

ILI data were obtained from the Texas Department of State Health Services website at the following URLs:

- www.dshs.texas.gov/idcu/disease/influenza/surveillance/2013/
- www.dshs.texas.gov/idcu/disease/influenza/surveillance/2014/
- www.dshs.texas.gov/idcu/disease/influenza/surveillance/2015/

## Wisconsin

ILI data were provided by Thomas E. Haupt of Wisconsin Department of Health Services.
