# Supplementary material for: Surveilling Influenza Incidence With Centers for Disease Control and Prevention Web Traffic Data: Demonstration Using a Novel Dataset
Source: J Med Internet Res. 2020 Jul 3;22(7):e14337. doi: 10.2196/14337 (PMC7367534; doi:10.2196/14337)
Supplement: Multimedia Appendix 5 [file jmir_v22i7e14337_app5.docx]

## Appendix E: Census Divisions

This section lists the states in each of the U.S. census divisions.

## East North Central

- Illinois
- Indiana
- Michigan
- Ohio
- Wisconsin

## East South Central

- Alabama
- Kentucky
- Mississippi
- Tennessee

## Middle Atlantic

- New Jersey
- New York
- Pennsylvania

## Mountain

- Arizona
- Colorado
- Idaho
- Montana
- Nevada
- New Mexico
- Utah
- Wyoming

## New England

- Connecticut
- Maine
- Massachusetts
- New Hampshire
- Rhode Island
- Vermont

## Pacific

- Alaska
- California
- Hawaii
- Oregon
- Washington

## South Atlantic

- Delaware
- Florida
- Georgia
- Maryland
- North Carolina
- South Carolina
- Virginia
- West Virginia

## West North Central

- Iowa
- Kansas
- Minnesota
- Missouri
- Nebraska
- North Dakota
- South Dakota

## West South Central

- Arkansas
- Louisiana
- Oklahoma
- Texas
