# Supplementary material for: Surveilling Influenza Incidence With Centers for Disease Control and Prevention Web Traffic Data: Demonstration Using a Novel Dataset
Source: J Med Internet Res. 2020 Jul 3;22(7):e14337. doi: 10.2196/14337 (PMC7367534; doi:10.2196/14337)
Supplement: Multimedia Appendix 6 [file jmir_v22i7e14337_app6.docx]

## Appendix F: Additional Results

This section contains results from models not included in the manuscript. Because these models were not included in the manuscript, not all metrics were calculated for each model. Models are listed in the tables from highest to lowest $r^{2}\boldsymbol{.}$Models with $r^{2}$ > 0.7 are shaded in green. Models with $r^{2}$ < 0.2 are shaded in orange.

## National Models

| **Pages Used in Model** | **Season(s)** | **Shift** | $\boldsymbol{r}^{\boldsymbol{2}}$ |
| --- | --- | --- | --- |
| FluView, Symptoms, Treatment | 2015–2016 | None | 0.891 |
| All | 2012–2013 | 1 Week | 0.889 |
| FluView, Symptoms, Treatment | 2012–2013 | 1 Week | 0.856 |
| All | 2012–2013 | None | 0.853 |
| All | 2015–2016 | None | 0.834 |
| FluView, Symptoms, Treatment | 2015–2016 | 1 Week | 0.784 |
| All | 2015–2016 | 1 Week | 0.762 |
| FluView, Symptoms, Treatment | 2014–2015 | None | 0.567 |
| FluView, Symptoms, Treatment | 2014–2015 | 1 Week | 0.453 |
| All | 2014–2015 | None | 0.327 |
| All | 2014–2015 | 1 Week | 0.296 |
| All | 2014–2016  (2 seasons) | None | 0.251 |
| All | 2013–2015  (2 seasons) | None | 0.241 |
| FluView, Symptoms, Treatment | 2013–2014 | None | 0.236 |
| FluView, Symptoms, Treatment | 2013–2014 | 1 Week | 0.191 |
| All | All | 1 Week | 0.166 |
| All | All | None | 0.142 |
| All | 2012–2014  (2 seasons) | None | 0.061 |
| All | 2013–2014 | None | 0.052 |
| All | 2013–2014 | 1 Week | 0.014 |

## Census Division Models

## *East North Central*

| **Pages Used in Model** | **Season(s)** | **Shift** | $\boldsymbol{r}^{\boldsymbol{2}}$ |
| --- | --- | --- | --- |
| FluView, Symptoms, Treatment | 2012–2013 | None | 0.895 |
| All | 2012–2013 | 1 Week | 0.855 |
| All | 2012–2013 | None | 0.806 |
| All | 2015–2016 | 1 Week | 0.772 |
| FluView, Symptoms, Treatment | 2015–2016 | None | 0.762 |
| All | 2015–2016 | None | 0.748 |
| FluView, Symptoms, Treatment | 2015–2016 | 1 Week | 0.728 |
| FluView, Symptoms, Treatment | 2014–2015 | None | 0.509 |
| FluView, Symptoms, Treatment | 2014–2015 | 1 Week | 0.421 |
| All | 2014–2015 | 1 Week | 0.284 |
| All | 2014–2015 | None | 0.271 |
| FluView, Symptoms, Treatment | 2013–2014 | None | 0.225 |
| FluView, Symptoms, Treatment | 2013–2014 | 1 Week | 0.191 |
| All | All | 1 Week | 0.134 |
| All | All | None | 0.105 |
| All | 2013–2014 | None | 0.042 |
| All | 2013–2014 | 1 Week | 0.013 |

## *East South Central*

| **Pages Used in Model** | **Season(s)** | **Shift** | $\boldsymbol{r}^{\boldsymbol{2}}$ |
| --- | --- | --- | --- |
| FluView, Symptoms, Treatment | 2012–2013 | 1 Week | 0.759 |
| All | 2015–2016 | 1 Week | 0.748 |
| FluView, Symptoms, Treatment | 2015–2016 | None | 0.745 |
| FluView, Symptoms, Treatment | 2012–2013 | None | 0.682 |
| All | 2015–2016 | None | 0.652 |
| All | 2012–2013 | 1 Week | 0.609 |
| All | 2012–2013 | None | 0.525 |
| FluView, Symptoms, Treatment | 2014–2015 | None | 0.316 |
| FluView, Symptoms, Treatment | 2014–2015 | 1 Week | 0.254 |
| FluView, Symptoms, Treatment | 2013–2014 | None | 0.139 |
| FluView, Symptoms, Treatment | 2013–2014 | 1 Week | 0.118 |
| All | All | 1 Week | 0.104 |
| All | All | None | 0.080 |
| All | 2014–2015 | None | 0.037 |
| All | 2014–2015 | 1 Week | 0.036 |
| All | 2013–2014 | 1 Week | 0.018 |
| All | 2013–2014 | None | 0.003 |

## *Middle Atlantic*

| **Pages Used in Model** | **Season(s)** | **Shift** | $\boldsymbol{r}^{\boldsymbol{2}}$ |
| --- | --- | --- | --- |
| FluView, Symptoms, Treatment | 2015–2016 | 1 Week | 0.855 |
| All | 2015–2016 | 1 Week | 0.801 |
| All | 2015–2016 | None | 0.781 |
| All | 2012–2013 | 1 Week | 0.779 |
| All | 2012–2013 | None | 0.770 |
| FluView, Symptoms, Treatment | 2012–2013 | None | 0.770 |
| FluView, Symptoms, Treatment | 2012–2013 | 1 Week | 0.733 |
| FluView, Symptoms, Treatment | 2013–2014 | None | 0.576 |
| FluView, Symptoms, Treatment | 2013–2014 | 1 Week | 0.537 |
| FluView, Symptoms, Treatment | 2014–2015 | None | 0.512 |
| FluView, Symptoms, Treatment | 2014–2015 | 1 Week | 0.456 |
| All | 2014–2015 | None | 0.292 |
| All | 2014–2015 | 1 Week | 0.278 |
| All | 2013–2014 | None | 0.211 |
| All | All | 1 Week | 0.144 |
| All | 2013–2014 | 1 Week | 0.139 |
| All | All | None | 0.118 |

## *Mountain*

| **Pages Used in Model** | **Season(s)** | **Shift** | $\boldsymbol{r}^{\boldsymbol{2}}$ |
| --- | --- | --- | --- |
| FluView, Symptoms, Treatment | 2015–2016 | None | 0.874 |
| All | 2012–2013 | None | 0.857 |
| All | 2012–2013 | 1 Week | 0.848 |
| All | 2012–2013 | None | 0.842 |
| FluView, Symptoms, Treatment | 2012–2013 | 1 Week | 0.817 |
| All | 2015–2016 | 1 Week | 0.768 |
| FluView, Symptoms, Treatment | 2015–2016 | 1 Week | 0.768 |
| FluView, Symptoms, Treatment | 2014–2015 | None | 0.523 |
| FluView, Symptoms, Treatment | 2014–2015 | 1 Week | 0.478 |
| All | 2014–2015 | None | 0.211 |
| FluView, Symptoms, Treatment | 2013–2014 | None | 0.200 |
| All | 2014–2015 | 1 Week | 0.199 |
| All | All | 1 Week | 0.183 |
| FluView, Symptoms, Treatment | 2013–2014 | 1 Week | 0.176 |
| All | All | None | 0.168 |
| All | 2013–2014 | None | 0.009 |
| All | 2013–2014 | 1 Week | 0.001 |

## *New England*

| **Pages Used in Model** | **Season(s)** | **Shift** | $\boldsymbol{r}^{\boldsymbol{2}}$ |
| --- | --- | --- | --- |
| FluView, Symptoms, Treatment | 2015–2016 | 1 Week | 0.869 |
| All | 2015–2016 | None | 0.816 |
| All | 2015–2016 | 1 Week | 0.785 |
| All | 2012–2013 | None | 0.767 |
| All | 2012–2013 | 1 Week | 0.714 |
| FluView, Symptoms, Treatment | 2012–2013 | None | 0.695 |
| FluView, Symptoms, Treatment | 2012–2013 | 1 Week | 0.639 |
| FluView, Symptoms, Treatment | 2013–2014 | 1 Week | 0.583 |
| FluView, Symptoms, Treatment | 2013–2014 | None | 0.574 |
| FluView, Symptoms, Treatment | 2014–2015 | None | 0.488 |
| FluView, Symptoms, Treatment | 2014–2015 | 1 Week | 0.472 |
| All | 2013–2014 | None | 0.373 |
| All | 2013–2014 | 1 Week | 0.369 |
| All | 2014–2015 | None | 0.352 |
| All | 2014–2015 | 1 Week | 0.335 |
| All | All | 1 Week | 0.228 |
| All | All | None | 0.194 |

## *Pacific*

| **Pages Used in Model** | **Season(s)** | **Shift** | $\boldsymbol{r}^{\boldsymbol{2}}$ |
| --- | --- | --- | --- |
| FluView, Symptoms, Treatment | 2015–2016 | None | 0.807 |
| All | 2015–2016 | None | 0.789 |
| FluView, Symptoms, Treatment | 2012–2013 | 1 Week | 0.718 |
| All | 2015–2016 | 1 Week | 0.644 |
| FluView, Symptoms, Treatment | 2015–2016 | 1 Week | 0.635 |
| All | 2012–2013 | 1 Week | 0.616 |
| All | 2012–2013 | None | 0.485 |
| FluView, Symptoms, Treatment | 2014–2015 | None | 0.314 |
| FluView, Symptoms, Treatment | 2014–2015 | 1 Week | 0.286 |
| FluView, Symptoms, Treatment | 2013–2014 | None | 0.104 |
| FluView, Symptoms, Treatment | 2013–2014 | 1 Week | 0.098 |
| All | All | 1 Week | 0.072 |
| All | All | None | 0.062 |
| All | 2014–2015 | None | 0.018 |
| All | 2014–2015 | 1 Week | 0.015 |
| All | 2013–2014 | None | 0.004 |
| All | 2013–2014 | 1 Week | 0.002 |

## *South Atlantic*

| **Pages Used in Model** | **Season(s)** | **Shift** | $\boldsymbol{r}^{\boldsymbol{2}}$ |
| --- | --- | --- | --- |
| FluView, Symptoms, Treatment | 2015–2016 | 1 Week | 0.851 |
| All | 2015–2016 | None | 0.780 |
| All | 2015–2016 | 1 Week | 0.775 |
| FluView, Symptoms, Treatment | 2012–2013 | 1 Week | 0.689 |
| FluView, Symptoms, Treatment | 2012–2013 | None | 0.629 |
| All | 2012–2013 | 1 Week | 0.588 |
| All | 2012–2013 | None | 0.507 |
| FluView, Symptoms, Treatment | 2014–2015 | None | 0.409 |
| FluView, Symptoms, Treatment | 2014–2015 | 1 Week | 0.329 |
| All | 2014–2015 | 1 Week | 0.242 |
| All | 2014–2015 | None | 0.220 |
| FluView, Symptoms, Treatment | 2013–2014 | None | 0.215 |
| FluView, Symptoms, Treatment | 2013–2014 | 1 Week | 0.188 |
| All | All | 1 Week | 0.150 |
| All | All | None | 0.127 |
| All | 2013–2014 | None | 0.088 |
| All | 2013–2014 | 1 Week | 0.057 |

## *West North Central*

| **Pages Used in Model** | **Season(s)** | **Shift** | $\boldsymbol{r}^{\boldsymbol{2}}$ |
| --- | --- | --- | --- |
| All | 2012–2013 | None | 0.927 |
| FluView, Symptoms, Treatment | 2012–2013 | 1 Week | 0.925 |
| All | 2012–2013 | 1 Week | 0.918 |
| FluView, Symptoms, Treatment | 2014–2015 | None | 0.719 |
| FluView, Symptoms, Treatment | 2015–2016 | None | 0.658 |
| FluView, Symptoms, Treatment | 2014–2015 | 1 Week | 0.648 |
| All | 2015–2016 | None | 0.647 |
| All | 2015–2016 | 1 Week | 0.638 |
| FluView, Symptoms, Treatment | 2015–2016 | 1 Week | 0.606 |
| All | 2014–2015 | None | 0.536 |
| All | 2014–2015 | 1 Week | 0.527 |
| FluView, Symptoms, Treatment | 2013–2014 | None | 0.305 |
| FluView, Symptoms, Treatment | 2013–2014 | 1 Week | 0.267 |
| All | All | 1 Week | 0.197 |
| All | All | None | 0.157 |
| All | 2013–2014 | None | 0.148 |
| All | 2013–2014 | 1 Week | 0.089 |

## *West South Central*

| **Pages Used in Model** | **Season(s)** | **Shift** | $\boldsymbol{r}^{\boldsymbol{2}}$ |
| --- | --- | --- | --- |
| All | 2012–2013 | None | 0.784 |
| All | 2012–2013 | 1 Week | 0.773 |
| FluView, Symptoms, Treatment | 2012–2013 | 1 Week | 0.732 |
| FluView, Symptoms, Treatment | 2014–2015 | None | 0.541 |
| FluView, Symptoms, Treatment | 2014–2015 | 1 Week | 0.436 |
| FluView, Symptoms, Treatment | 2015–2016 | None | 0.386 |
| All | 2015–2016 | None | 0.367 |
| FluView, Symptoms, Treatment | 2015–2016 | 1 Week | 0.193 |
| FluView, Symptoms, Treatment | 2013–2014 | None | 0.181 |
| All | 2015–2016 | 1 Week | 0.176 |
| All | 2014–2015 | None | 0.171 |
| All | 2014–2015 | 1 Week | 0.153 |
| FluView, Symptoms, Treatment | 2013–2014 | 1 Week | 0.145 |
| All | All | 1 Week | 0.112 |
| All | All | None | 0.109 |
| All | 2013–2014 | 1 Week | 0.005 |
| All | 2013–2014 | None | 0.00 |

## State Models

## *California*

| **Pages Used in Model** | **Season(s)** | **Shift** | $\boldsymbol{r}^{\boldsymbol{2}}$ |
| --- | --- | --- | --- |
| FluView | 2013–2014 | None | 0.734 |
| All | 2012–2013 | 2 Week | 0.723 |
| FluView, Symptoms, Treatment | 2013–2014 | 1 Week | 0.704 |
| Treatment | 2013–2014 | None | 0.673 |
| Antivirals | 2014–2015 | None | 0.639 |
| Antivirals, Symptoms | 2012–2013 | 1 Week | 0.632 |
| FluView, Symptoms, Treatment | 2013–2014 | None | 0.612 |
| Antivirals, Symptoms | 2013–2014 | 1 Week | 0.600 |
| All | 2012–2013 | None | 0.565 |
| Vaccine | 2013–2014 | None | 0.544 |
| Antivirals, Symptoms | 2015–2016 | None | 0.538 |
| FluView, Symptoms, Treatment | 2015–2016 | None | 0.538 |
| Symptoms | 2015–2016 | None | 0.537 |
| High Risk Complications | 2014–2015 | None | 0.511 |
| Antivirals, Symptoms | 2013–2014 | None | 0.503 |
| Treat Flu | 2013–2014 | None | 0.501 |
| Treat Flu | 2012–2013 | None | 0.497 |
| Flu Basics | 2013–2014 | None | 0.494 |
| All | 2015–2016 | None | 0.489 |
| Symptoms | 2013–2014 | None | 0.486 |
| Key Facts, Prevention, Treat Flu, Vaccine | 2013–2014 | 1 Week | 0.456 |
| FluView, Symptoms, Treatment | 2012–2013 | None | 0.463 |
| Prevention | 2014–2015 | None | 0.420 |
| Antivirals, Symptoms | 2015–2016 | 1 Week | 0.420 |
| Treatment | 2015–2016 | None | 0.419 |
| Antivirals, Symptoms | 2012–2013 | None | 0.413 |
| Prevention | 2013–2014 | None | 0.407 |
| Symptoms | 2012–2013 | None | 0.395 |
| FluView | 2012–2013 | None | 0.376 |
| All | 2015–2016 | 1 Week | 0.370 |
| Vaccine | 2012–2013 | None | 0.368 |
| Treatment | 2012–2013 | None | 0.363 |
| Key Facts, Prevention, Treat Flu, Vaccine | 2013–2014 | None | 0.349 |
| Antivirals | 2012–2013 | None | 0.342 |
| FluView | 2014–2015 | None | 0.337 |
| Key Facts | 2013–2014 | None | 0.329 |
| Treatment | 2014–2015 | None | 0.315 |
| Vaccine | 2015–2016 | None | 0.311 |
| Key Facts, Prevention, Treat Flu, Vaccine | 2015–2016 | None | 0.309 |
| Key Facts | 2015–2016 | None | 0.303 |
| Key Facts, Prevention, Treat Flu, Vaccine | 2015–2016 | 1 Week | 0.293 |
| Antivirals | 2015–2016 | None | 0.289 |
| All | 2015–2016 | 2 Week | 0.288 |
| Flu Basics | 2012–2013 | None | 0.239 |
| Vaccine | 2014–2015 | None | 0.216 |
| FluView, Symptoms, Treatment | 2014–2015 | 1 Week | 0.188 |
| All | 2013–2014 | None | 0.171 |
| High Risk Complications | 2012–2013 | None | 0.164 |
| Key Facts | 2012–2013 | None | 0.140 |
| All | 2013–2014 | 1 Week | 0.130 |
| Antivirals, Symptoms | 2014–2015 | 1 Week | 0.114 |
| Treat Flu | 2014–2015 | None | 0.111 |
| FluView, Symptoms, Treatment | 2014–2015 | None | 0.106 |
| Symptoms | 2014–2015 | None | 0.104 |
| Flu Basics | 2015–2016 | None | 0.103 |
| Key Facts | 2014–2015 | None | 0.099 |
| Key Facts, Prevention, Treat Flu, Vaccine | 2014–2015 | 1 Week | 0.082 |
| High Risk Complications | 2013–2014 | None | 0.077 |
| Prevention | 2012–2013 | None | 0.071 |
| Antivirals, Symptoms | 2014–2015 | None | 0.066 |
| Antivirals | 2013–2014 | None | 0.064 |
| High Risk Complications | 2015–2016 | None | 0.059 |
| All | 2014–2015 | None | 0.055 |
| All | 2014–2015 | 1 Week | 0.053 |
| Key Facts, Prevention, Treat Flu, Vaccine | 2014–2015 | None | 0.048 |
| All | 2013–2014 | 2 Week | 0.036 |
| Treat Flu | 2015–2016 | None | 0.034 |
| Key Facts, Prevention, Treat Flu, Vaccine | 2012–2013 | None | 0.032 |
| FluView, Symptoms, Treatment | All | None | 0.030 |
| Key Facts, Prevention, Treat Flu, Vaccine | 2012–2013 | 1 Week | 0.017 |
| All | 2014–2015 | 2 Week | 0.014 |
| Prevention | 2015–2016 | None | 0.010 |
| FluView | 2015–2016 | None | 0.001 |
| All | All | None | 0.001 |
| Flu Basics | 2014–2015 | None | 0.000 |

## *Maine*

| **Pages Used in Model** | **Season(s)** | **Shift** | $\boldsymbol{r}^{\boldsymbol{2}}$ |
| --- | --- | --- | --- |
| High Risk Complications | 2012–2013 | None | 0.422 |
| FluView | 2012–2013 | None | 0.379 |
| Antivirals | 2014–2015 | None | 0.345 |
| Treatment | 2014–2015 | None | 0.331 |
| FluView, Symptoms, Treatment | 2013–2014 | 1 Week | 0.324 |
| FluView, Symptoms, Treatment | 2014–2015 | 1 Week | 0.314 |
| Key Facts | 2013–2014 | None | 0.296 |
| Antivirals, Symptoms | 2012–2013 | None | 0.296 |
| FluView, Symptoms, Treatment | 2012–2013 | None | 0.292 |
| All | 2014–2015 | 1 Week | 0.288 |
| Key Facts, Prevention, Treat Flu, Vaccine | 2013–2014 | None | 0.288 |
| Antivirals, Symptoms | 2014–2015 | 1 Week | 0.280 |
| FluView, Symptoms, Treatment | 2014–2015 | None | 0.278 |
| Antivirals, Symptoms | 2014–2015 | None | 0.253 |
| Antivirals, Symptoms | 2013–2014 | 1 Week | 0.248 |
| Symptoms | 2014–2015 | None | 0.236 |
| Treatment | 2013–2014 | None | 0.232 |
| FluView, Symptoms, Treatment | 2013–2014 | None | 0.218 |
| FluView | 2013–2014 | None | 0.204 |
| All | 2014–2015 | None | 0.203 |
| Key Facts, Prevention, Treat Flu, Vaccine | 2013–2014 | 1 Week | 0.202 |
| All | 2014–2015 | 2 Week | 0.192 |
| All | 2012–2013 | 2 Week | 0.185 |
| All | 2012–2013 | None | 0.177 |
| Vaccine | 2013–2014 | None | 0.170 |
| Symptoms | 2012–2013 | None | 0.152 |
| Key Facts | 2014–2015 | None | 0.151 |
| FluView, Symptoms, Treatment | 2012–2013 | 1 Week | 0.147 |
| Antivirals, Symptoms | 2013–2014 | None | 0.144 |
| Key Facts, Prevention, Treat Flu, Vaccine | 2014–2015 | None | 0.144 |
| Symptoms | 2013–2014 | None | 0.137 |
| Prevention | 2013–2014 | None | 0.132 |
| Treatment | 2012–2013 | None | 0.129 |
| Key Facts, Prevention, Treat Flu, Vaccine | 2014–2015 | 1 Week | 0.119 |
| Flu Basics | 2013–2014 | None | 0.111 |
| High Risk Complications | 2014–2015 | None | 0.109 |
| Prevention | 2012–2013 | None | 0.088 |
| Flu Basics | 2014–2015 | None | 0.087 |
| Antivirals | 2013–2014 | None | 0.085 |
| High Risk Complications | 2013–2014 | None | 0.085 |
| Antivirals, Symptoms | 2012–2013 | 1 Week | 0.083 |
| All | 2013–2014 | 2 Week | 0.079 |
| All | 2013–2014 | 1 Week | 0.074 |
| All | 2012–2013 | 1 Week | 0.072 |
| Vaccine | 2012–2013 | None | 0.069 |
| Key Facts, Prevention, Treat Flu, Vaccine | 2012–2013 | 1 Week | 0.060 |
| Flu Basics | 2012–2013 | None | 0.041 |
| Prevention | 2014–2015 | None | 0.038 |
| All | All | None | 0.034 |
| Key Facts | 2012–2013 | None | 0.033 |
| Key Facts, Prevention, Treat Flu, Vaccine | 2012–2013 | None | 0.033 |
| Key Facts, Prevention, Treat Flu, Vaccine | 2015–2016 | 1 Week | 0.029 |
| FluView, Symptoms, Treatment | All | None | 0.026 |
| FluView | 2014–2015 | None | 0.025 |
| Symptoms | 2014–2015 | None | 0.025 |
| Antivirals, Symptoms | 2015–2016 | None | 0.025 |
| FluView, Symptoms, Treatment | 2015–2016 | None | 0.022 |
| FluView | 2014–2015 | None | 0.019 |
| All | 2015–2016 | None | 0.018 |
| FluView, Symptoms, Treatment | 2015–2016 | 1 Week | 0.016 |
| Antivirals, Symptoms | 2015–2016 | 1 Week | 0.013 |
| Key Facts, Prevention, Treat Flu, Vaccine | 2015–2016 | None | 0.012 |
| Key Facts | 2014–2015 | None | 0.009 |
| All | 2015–2016 | 1 Week | 0.009 |
| Prevention | 2014–2015 | None | 0.006 |
| Treat Flu | 2014–2015 | None | 0.005 |
| All | 2015–2016 | 2 Week | 0.005 |
| Vaccine | 2014–2015 | None | 0.004 |
| Antivirals | 2014–2015 | None | 0.004 |
| All | 2013–2014 | None | 0.003 |
| Treat Flu | 2013–2014 | None | 0.003 |
| Vaccine | 2014–2015 | None | 0.002 |
| Flu Basics | 2014–2015 | None | 0.001 |
| Treatment | 2014–2015 | None | 0.001 |
| Treat Flu | 2014–2015 | None | 0.001 |
| Treat Flu | 2012–2013 | None | 0.000 |
| High Risk Complications | 2014–2015 | None | 0.000 |

## *Missouri*

| **Pages Used in Model** | **Season(s)** | **Shift** | $\boldsymbol{r}^{\boldsymbol{2}}$ |
| --- | --- | --- | --- |
| Antivirals, Symptoms | 2012–2013 | 1 Week | 0.735 |
| All | 2012–2013 | 2 Week | 0.703 |
| FluView, Symptoms, Treatment | 2012–2013 | None | 0.582 |
| FluView, Symptoms, Treatment | 2014–2015 | None | 0.575 |
| Symptoms | 2014–2015 | None | 0.553 |
| Antivirals, Symptoms | 2014–2015 | None | 0.549 |
| Symptoms | 2012–2013 | None | 0.548 |
| FluView, Symptoms, Treatment | 2014–2015 | 1 Week | 0.548 |
| Antivirals, Symptoms | 2012–2013 | None | 0.541 |
| FluView | 2014–2015 | None | 0.531 |
| Antivirals, Symptoms | 2014–2015 | 1 Week | 0.519 |
| All | 2012–2013 | None | 0.510 |
| Treatment | 2014–2015 | None | 0.448 |
| Vaccine | 2013–2014 | None | 0.430 |
| All | 2015–2016 | None | 0.391 |
| FluView | 2012–2013 | None | 0.385 |
| FluView | 2013–2014 | None | 0.380 |
| All | 2015–2016 | 1 Week | 0.370 |
| High Risk Complications | 2012–2013 | None | 0.335 |
| Treatment | 2012–2013 | None | 0.325 |
| All | 2014–2015 | None | 0.322 |
| All | 2014–2015 | 1 Week | 0.318 |
| All | 2014–2015 | 2 Week | 0.293 |
| Antivirals, Symptoms | 2015–2016 | None | 0.278 |
| FluView, Symptoms, Treatment | 2015–2016 | None | 0.263 |
| Key Facts, Prevention, Treat Flu, Vaccine | 2012–2013 | 1 Week | 0.263 |
| Flu Basics | 2012–2013 | None | 0.247 |
| All | 2015–2016 | 2 Week | 0.243 |
| Vaccine | 2014–2015 | None | 0.238 |
| Antivirals, Symptoms | 2015–2016 | 1 Week | 0.235 |
| Flu Basics | 2014–2015 | None | 0.229 |
| Symptoms | 2015–2016 | None | 0.227 |
| Key Facts, Prevention, Treat Flu, Vaccine | 2014–2015 | 1 Week | 0.227 |
| Key Facts, Prevention, Treat Flu, Vaccine | 2014–2015 | None | 0.226 |
| Key Facts | 2014–2015 | None | 0.225 |
| FluView, Symptoms, Treatment | 2015–2016 | 1 Week | 0.222 |
| Antivirals | 2015–2016 | None | 0.194 |
| FluView, Symptoms, Treatment | 2013–2014 | None | 0.188 |
| Antivirals | 2012–2013 | None | 0.183 |
| Key Facts, Prevention, Treat Flu, Vaccine | 2013–2014 | 1 Week | 0.148 |
| Key Facts, Prevention, Treat Flu, Vaccine | 2013–2014 | None | 0.148 |
| FluView, Symptoms, Treatment | 2013–2014 | 1 Week | 0.136 |
| Treatment | 2013–2014 | None | 0.115 |
| Key Facts | 2013–2014 | None | 0.109 |
| Treat Flu | 2012–2013 | None | 0.104 |
| Antivirals | 2014–2015 | None | 0.087 |
| Key Facts, Prevention, Treat Flu, Vaccine | 2012–2013 | None | 0.085 |
| Treatment | 2015–2016 | None | 0.083 |
| Treat Flu | 2015–2016 | None | 0.081 |
| Antivirals, Symptoms | 2013–2014 | 1 Week | 0.078 |
| FluView, Symptoms, Treatment | All | None | 0.071 |
| Key Facts | 2012–2013 | None | 0.068 |
| Symptoms | 2013–2014 | None | 0.060 |
| Antivirals, Symptoms | 2013–2014 | None | 0.057 |
| Vaccine | 2012–2013 | None | 0.038 |
| Treat Flu | 2014–2015 | None | 0.035 |
| Vaccine | 2015–2016 | None | 0.025 |
| Flu Basics | 2013–2014 | None | 0.024 |
| All | All | None | 0.022 |
| Prevention | 2014–2015 | None | 0.017 |
| Prevention | 2015–2016 | None | 0.015 |
| Antivirals | 2013–2014 | None | 0.014 |
| Prevention | 2012–2013 | None | 0.012 |
| Treat Flu | 2013–2014 | None | 0.009 |
| FluView | 2015–2016 | None | 0.009 |
| High Risk Complications | 2015–2016 | None | 0.007 |
| All | 2013–2014 | 2 Week | 0.007 |
| Key Facts, Prevention, Treat Flu, Vaccine | 2015–2016 | None | 0.006 |
| High Risk Complications | 2013–2014 | None | 0.005 |
| Key Facts | 2015–2016 | None | 0.005 |
| Prevention | 2013–2014 | None | 0.004 |
| Key Facts, Prevention, Treat Flu, Vaccine | 2015–2016 | 1 Week | 0.004 |
| Flu Basics | 2015–2016 | None | 0.003 |
| All | 2013–2014 | 1 Week | 0.003 |
| All | 2013–2014 | None | 0.002 |
| High Risk Complications | 2014–2015 | None | 0.001 |

## *New Jersey*

| **Pages Used in Model** | **Season(s)** | **Shift** | $\boldsymbol{r}^{\boldsymbol{2}}$ |
| --- | --- | --- | --- |
| FluView, Symptoms, Treatment | 2012–2013 | None | 0.820 |
| Antivirals, Symptoms | 2012–2013 | None | 0.811 |
| All | 2012–2013 | 2 Week | 0.809 |
| Symptoms | 2012–2013 | None | 0.784 |
| FluView, Symptoms, Treatment | 2012–2013 | 1 Week | 0.754 |
| Treatment | 2015–2016 | None | 0.720 |
| FluView, Symptoms, Treatment | 2015–2016 | None | 0.716 |
| Key Facts, Prevention, Treat Flu, Vaccine | 2015–2016 | None | 0.707 |
| Antivirals, Symptoms | 2012–2013 | 1 Week | 0.704 |
| FluView, Symptoms, Treatment | 2015–2016 | 1 Week | 0.703 |
| Symptoms | 2012–2013 | 1 Week | 0.701 |
| Symptoms | 2015–2016 | None | 0.697 |
| Antivirals, Symptoms | 2015–2016 | None | 0.697 |
| Key Facts | 2015–2016 | None | 0.691 |
| Key Facts, Prevention, Treat Flu, Vaccine | 2015–2016 | 1 Week | 0.691 |
| Symptoms | 2012–2013 | 2 Week | 0.688 |
| Antivirals, Symptoms | 2015–2016 | 1 Week | 0.686 |
| All | 2012–2013 | None | 0.668 |
| FluView | 2012–2013 | 2 Week | 0.661 |
| Flu Basics | 2012–2013 | 1 Week | 0.656 |
| Flu Basics | 2012–2013 | None | 0.598 |
| Flu Basics | 2012–2013 | 2 Week | 0.596 |
| Antivirals | 2012–2013 | None | 0.578 |
| FluView | 2012–2013 | 1 Week | 0.559 |
| Antivirals | 2012–2013 | 1 Week | 0.552 |
| All | 2015–2016 | None | 0.533 |
| Vaccine | 2015–2016 | None | 0.530 |
| All | 2015–2016 | 1 Week | 0.508 |
| Treat Flu | 2012–2013 | 2 Week | 0.462 |
| FluView | 2012–2013 | None | 0.458 |
| Key Facts, Prevention, Treat Flu, Vaccine | 2012–2013 | 1 Week | 0.448 |
| Antivirals | 2015–2016 | None | 0.415 |
| All | 2015–2016 | 2 Week | 0.411 |
| Flu Basics | 2015–2016 | None | 0.402 |
| Antivirals | 2012–2013 | 2 Week | 0.400 |
| FluView, Symptoms, Treatment | All | None | 0.399 |
| Key Facts | 2012–2013 | 2 Week | 0.389 |
| High Risk Complications | 2012–2013 | None | 0.388 |
| Antivirals | 2014–2015 | None | 0.379 |
| High Risk Complications | 2012–2013 | 1 Week | 0.378 |
| Treatment | 2012–2013 | 2 Week | 0.371 |
| Treatment | 2014–2015 | None | 0.354 |
| Treatment | 2013–2014 | None | 0.341 |
| Key Facts | 2013–2014 | None | 0.339 |
| Key Facts, Prevention, Treat Flu, Vaccine | 2013–2014 | None | 0.339 |
| FluView, Symptoms, Treatment | 2013–2014 | 1 Week | 0.338 |
| FluView, Symptoms, Treatment | 2013–2014 | None | 0.329 |
| Antivirals, Symptoms | 2013–2014 | 1 Week | 0.315 |
| FluView, Symptoms, Treatment | 2014–2015 | None | 0.306 |
| High Risk Complications | 2012–2013 | 2 Week | 0.303 |
| Key Facts, Prevention, Treat Flu, Vaccine | 2013–2014 | 1 Week | 0.289 |
| Flu Basics | 2013–2014 | None | 0.287 |
| Antivirals, Symptoms | 2013–2014 | None | 0.281 |
| Symptoms | 2013–2014 | None | 0.280 |
| Antivirals, Symptoms | 2014–2015 | None | 0.276 |
| Symptoms | 2014–2015 | None | 0.264 |
| FluView | 2013–2014 | None | 0.262 |
| Treatment | 2012–2013 | None | 0.246 |
| Treatment | 2012–2013 | 1 Week | 0.246 |
| All | All | None | 0.234 |
| Vaccine | 2014–2015 | None | 0.231 |
| Vaccine | 2013–2014 | None | 0.229 |
| FluView, Symptoms, Treatment | 2014–2015 | 1 Week | 0.222 |
| Prevention | 2013–2014 | None | 0.221 |
| Key Facts | 2012–2013 | 1 Week | 0.207 |
| Treat Flu | 2012–2013 | None | 0.195 |
| Key Facts | 2012–2013 | None | 0.194 |
| Antivirals, Symptoms | 2014–2015 | 1 Week | 0.181 |
| Treat Flu | 2012–2013 | 1 Week | 0.181 |
| High Risk Complications | 2014–2015 | None | 0.180 |
| Treat Flu | 2013–2014 | None | 0.155 |
| FluView | 2014–2015 | None | 0.129 |
| Key Facts, Prevention, Treat Flu, Vaccine | 2014–2015 | None | 0.121 |
| All | 2013–2014 | None | 0.120 |
| All | 2014–2015 | None | 0.119 |
| Key Facts | 2014–2015 | None | 0.117 |
| All | 2013–2014 | 1 Week | 0.108 |
| Prevention | 2015–2016 | None | 0.096 |
| Key Facts, Prevention, Treat Flu, Vaccine | 2014–2015 | 1 Week | 0.095 |
| All | 2014–2015 | 1 Week | 0.089 |
| High Risk Complications | 2013–2014 | None | 0.081 |
| High Risk Complications | 2015–2016 | None | 0.077 |
| Treat Flu | 2014–2015 | None | 0.070 |
| Prevention | 2014–2015 | None | 0.059 |
| Key Facts, Prevention, Treat Flu, Vaccine | 2012–2013 | None | 0.056 |
| All | 2014–2015 | 2 Week | 0.048 |
| FluView | 2015–2016 | None | 0.043 |
| Prevention | 2012–2013 | 1 Week | 0.035 |
| Vaccine | 2012–2013 | 1 Week | 0.032 |
| Antivirals | 2013–2014 | None | 0.031 |
| Prevention | 2012–2013 | None | 0.025 |
| Vaccine | 2012–2013 | None | 0.019 |
| Prevention | 2012–2013 | 2 Week | 0.019 |
| All | 2013–2014 | 2 Week | 0.017 |
| Flu Basics | 2014–2015 | None | 0.011 |
| Vaccine | 2012–2013 | 2 Week | 0.010 |
| Treat Flu | 2015–2016 | None | 0.000 |

## *New Mexico*

| **Pages Used in Model** | **Season(s)** | **Shift** | $\boldsymbol{r}^{\boldsymbol{2}}$ |
| --- | --- | --- | --- |
| Antivirals, Symptoms | 2015–2016 | 1 Week | 0.765 |
| FluView, Symptoms, Treatment | 2015–2016 | 1 Week | 0.758 |
| FluView, Symptoms, Treatment | 2012–2013 | None | 0.700 |
| Antivirals, Symptoms | 2012–2013 | None | 0.685 |
| Antivirals, Symptoms | 2012–2013 | 1 Week | 0.632 |
| Symptoms | 2012–2013 | None | 0.621 |
| All | 2015–2016 | 2 Week | 0.594 |
| FluView, Symptoms, Treatment | 2012–2013 | 1 Week | 0.594 |
| FluView | 2012–2013 | None | 0.555 |
| Antivirals | 2014–2015 | None | 0.486 |
| All | 2015–2016 | None | 0.484 |
| Flu Basics | 2012–2013 | None | 0.475 |
| FluView | 2014–2015 | None | 0.464 |
| Treatment | 2014–2015 | None | 0.446 |
| FluView, Symptoms, Treatment | 2014–2015 | None | 0.441 |
| Antivirals, Symptoms | 2015–2016 | None | 0.436 |
| Symptoms | 2015–2016 | None | 0.435 |
| FluView, Symptoms, Treatment | 2015–2016 | None | 0.433 |
| All | 2012–2013 | 1 Week | 0.429 |
| All | 2012–2013 | None | 0.417 |
| FluView | 2013–2014 | None | 0.395 |
| FluView, Symptoms, Treatment | 2014–2015 | 1 Week | 0.375 |
| Key Facts, Prevention, Treat Flu, Vaccine | 2015–2016 | 1 Week | 0.370 |
| Antivirals | 2012–2013 | None | 0.363 |
| All | 2012–2013 | 2 Week | 0.361 |
| Antivirals, Symptoms | 2014–2015 | None | 0.326 |
| Symptoms | 2014–2015 | None | 0.288 |
| Antivirals, Symptoms | 2014–2015 | 1 Week | 0.259 |
| Treatment | 2015–2016 | None | 0.254 |
| Key Facts, Prevention, Treat Flu, Vaccine | 2014–2015 | 1 Week | 0.219 |
| Key Facts, Prevention, Treat Flu, Vaccine | 2014–2015 | None | 0.217 |
| Key Facts | 2014–2015 | None | 0.214 |
| All | 2014–2015 | None | 0.201 |
| Vaccine | 2013–2014 | None | 0.194 |
| All | 2014–2015 | 1 Week | 0.184 |
| Vaccine | 2014–2015 | None | 0.155 |
| Key Facts, Prevention, Treat Flu, Vaccine | 2013–2014 | 1 Week | 0.143 |
| Key Facts | 2015–2016 | None | 0.136 |
| Key Facts, Prevention, Treat Flu, Vaccine | 2015–2016 | None | 0.133 |
| Key Facts, Prevention, Treat Flu, Vaccine | 2013–2014 | None | 0.124 |
| Key Facts | 2013–2014 | None | 0.114 |
| FluView, Symptoms, Treatment | 2013–2014 | 1 Week | 0.103 |
| Treatment | 2013–2014 | None | 0.101 |
| High Risk Complications | 2012–2013 | None | 0.092 |
| Vaccine | 2015–2016 | None | 0.086 |
| FluView, Symptoms, Treatment | All | None | 0.084 |
| FluView, Symptoms, Treatment | 2013–2014 | None | 0.081 |
| Treat Flu | 2013–2014 | None | 0.071 |
| Prevention | 2013–2014 | None | 0.055 |
| Antivirals | 2015–2016 | None | 0.041 |
| FluView | 2015–2016 | None | 0.033 |
| Prevention | 2015–2016 | None | 0.033 |
| All | 2013–2014 | 2 Week | 0.032 |
| Prevention | 2012–2013 | None | 0.030 |
| Treat Flu | 2015–2016 | None | 0.029 |
| Treat Flu | 2014–2015 | None | 0.028 |
| Antivirals, Symptoms | 2013–2014 | 1 Week | 0.028 |
| All | All | None | 0.027 |
| Flu Basics | 2015–2016 | None | 0.025 |
| All | 2014–2015 | 2 Week | 0.024 |
| High Risk Complications | 2015–2016 | None | 0.020 |
| All | 2013–2014 | None | 0.016 |
| Treat Flu | 2012–2013 | None | 0.013 |
| Flu Basics | 2014–2015 | None | 0.013 |
| All | 2013–2014 | 1 Week | 0.012 |
| Antivirals, Symptoms | 2013–2014 | None | 0.012 |
| Symptoms | 2013–2014 | None | 0.011 |
| Antivirals | 2013–2014 | None | 0.007 |
| High Risk Complications | 2013–2014 | None | 0.006 |
| Treatment | 2012–2013 | None | 0.005 |
| Vaccine | 2012–2013 | None | 0.003 |
| Key Facts, Prevention, Treat Flu, Vaccine | 2012–2013 | 1 Week | 0.003 |
| Prevention | 2014–2015 | None | 0.001 |
| Key Facts | 2012–2013 | None | 0.000 |
| Flu Basics | 2013–2014 | None | 0.000 |
| High Risk Complications | 2014–2015 | None | 0.000 |
| Key Facts, Prevention, Treat Flu, Vaccine | 2012–2013 | None | 0.000 |

## *North Carolina*

| **Pages Used in Model** | **Season(s)** | **Shift** | $\boldsymbol{r}^{\boldsymbol{2}}$ |
| --- | --- | --- | --- |
| Antivirals, Symptoms | 2015–2016 | 1 Week | 0.776 |
| FluView | 2013–2014 | None | 0.722 |
| FluView, Symptoms, Treatment | 2015–2016 | None | 0.709 |
| Antivirals, Symptoms | 2015–2016 | None | 0.697 |
| Symptoms | 2015–2016 | None | 0.696 |
| All | 2015–2016 | 2 Week | 0.695 |
| FluView | 2012–2013 | None | 0.691 |
| All | 2015–2016 | None | 0.647 |
| Treatment | 2015–2016 | None | 0.514 |
| FluView, Symptoms, Treatment | 2012–2013 | 1 Week | 0.496 |
| FluView, Symptoms, Treatment | 2012–2013 | None | 0.495 |
| Key Facts, Prevention, Treat Flu, Vaccine | 2015–2016 | 1 Week | 0.487 |
| Vaccine | 2015–2016 | None | 0.453 |
| FluView | 2014–2015 | None | 0.447 |
| Key Facts, Prevention, Treat Flu, Vaccine | 2015–2016 | None | 0.442 |
| Key Facts | 2015–2016 | None | 0.438 |
| Antivirals | 2014–2015 | None | 0.428 |
| FluView, Symptoms, Treatment | 2014–2015 | None | 0.403 |
| Antivirals | 2015–2016 | None | 0.395 |
| Vaccine | 2014–2015 | None | 0.383 |
| Antivirals, Symptoms | 2014–2015 | None | 0.359 |
| FluView, Symptoms, Treatment | 2014–2015 | 1 Week | 0.348 |
| Symptoms | 2014–2015 | None | 0.345 |
| Vaccine | 2013–2014 | None | 0.323 |
| Antivirals, Symptoms | 2014–2015 | 1 Week | 0.296 |
| Treatment | 2012–2013 | None | 0.286 |
| Treatment | 2014–2015 | None | 0.283 |
| Key Facts | 2014–2015 | None | 0.258 |
| Symptoms | 2012–2013 | None | 0.252 |
| Key Facts, Prevention, Treat Flu, Vaccine | 2014–2015 | None | 0.252 |
| Key Facts, Prevention, Treat Flu, Vaccine | 2014–2015 | 1 Week | 0.237 |
| Treat Flu | 2012–2013 | None | 0.226 |
| All | 2012–2013 | 1 Week | 0.225 |
| Antivirals, Symptoms | 2012–2013 | None | 0.221 |
| Prevention | 2014–2015 | None | 0.220 |
| All | 2012–2013 | 2 Week | 0.220 |
| Antivirals, Symptoms | 2012–2013 | 1 Week | 0.208 |
| Key Facts, Prevention, Treat Flu, Vaccine | 2013–2014 | 1 Week | 0.187 |
| All | 2012–2013 | None | 0.185 |
| Flu Basics | 2012–2013 | None | 0.171 |
| FluView, Symptoms, Treatment | All | None | 0.168 |
| FluView, Symptoms, Treatment | 2013–2014 | 1 Week | 0.153 |
| Key Facts, Prevention, Treat Flu, Vaccine | 2013–2014 | None | 0.141 |
| Prevention | 2015–2016 | None | 0.140 |
| High Risk Complications | 2014–2015 | None | 0.139 |
| High Risk Complications | 2012–2013 | None | 0.126 |
| Key Facts | 2013–2014 | None | 0.118 |
| FluView, Symptoms, Treatment | 2013–2014 | None | 0.118 |
| Prevention | 2012–2013 | None | 0.109 |
| Treatment | 2013–2014 | None | 0.097 |
| Antivirals, Symptoms | 2013–2014 | 1 Week | 0.090 |
| Flu Basics | 2015–2016 | None | 0.067 |
| All | All | None | 0.067 |
| Symptoms | 2013–2014 | None | 0.054 |
| All | 2014–2015 | None | 0.053 |
| Treat Flu | 2015–2016 | None | 0.052 |
| Antivirals, Symptoms | 2013–2014 | None | 0.052 |
| Antivirals | 2012–2013 | None | 0.045 |
| Key Facts, Prevention, Treat Flu, Vaccine | 2012–2013 | None | 0.033 |
| FluView | 2015–2016 | None | 0.029 |
| High Risk Complications | 2013–2014 | None | 0.028 |
| Treat Flu | 2014–2015 | None | 0.028 |
| All | 2014–2015 | 1 Week | 0.021 |
| Flu Basics | 2014–2015 | None | 0.015 |
| Treat Flu | 2013–2014 | None | 0.013 |
| All | 2014–2015 | 2 Week | 0.012 |
| Vaccine | 2012–2013 | None | 0.011 |
| Antivirals | 2013–2014 | None | 0.011 |
| All | 2013–2014 | 1 Week | 0.010 |
| Key Facts | 2012–2013 | None | 0.007 |
| Prevention | 2013–2014 | None | 0.006 |
| All | 2013–2014 | 2 Week | 0.004 |
| All | 2013–2014 | None | 0.004 |
| High Risk Complications | 2015–2016 | None | 0.003 |
| Key Facts, Prevention, Treat Flu, Vaccine | 2012–2013 | 1 Week | 0.003 |
| Flu Basics | 2013–2014 | None | 0.001 |

## *Texas*

| **Pages Used in Model** | **Season(s)** | **Shift** | $\boldsymbol{r}^{\boldsymbol{2}}$ |
| --- | --- | --- | --- |
| All | 2012–2013 | None | 0.881 |
| FluView, Symptoms, Treatment | 2012–2013 | None | 0.881 |
| All | 2012–2013 | 2 Week | 0.863 |
| Symptoms | 2012–2013 | None | 0.825 |
| FluView, Symptoms, Treatment | 2012–2013 | 1 Week | 0.804 |
| Antivirals, Symptoms | 2012–2013 | None | 0.789 |
| FluView | 2012–2013 | None | 0.714 |
| Flu Basics | 2012–2013 | None | 0.682 |
| Antivirals, Symptoms | 2012–2013 | 1 Week | 0.679 |
| Treatment | 2014–2015 | None | 0.562 |
| High Risk Complications | 2012–2013 | None | 0.553 |
| Antivirals | 2014–2015 | None | 0.468 |
| Vaccine | 2013–2014 | None | 0.443 |
| FluView, Symptoms, Treatment | 2014–2015 | None | 0.442 |
| Vaccine | 2014–2015 | None | 0.423 |
| FluView, Symptoms, Treatment | 2014–2015 | 1 Week | 0.391 |
| Key Facts, Prevention, Treat Flu, Vaccine | 2015–2016 | None | 0.385 |
| Key Facts, Prevention, Treat Flu, Vaccine | 2012–2013 | 1 Week | 0.383 |
| Key Facts | 2015–2016 | None | 0.380 |
| Vaccine | 2015–2016 | None | 0.371 |
| Antivirals, Symptoms | 2014–2015 | None | 0.356 |
| FluView | 2014–2015 | None | 0.351 |
| Key Facts, Prevention, Treat Flu, Vaccine | 2015–2016 | 1 Week | 0.351 |
| Symptoms | 2014–2015 | None | 0.342 |
| Key Facts | 2012–2013 | None | 0.333 |
| Vaccine | 2012–2013 | None | 0.323 |
| Flu View | 2013–2014 | None | 0.305 |
| Antivirals, Symptoms | 2014–2015 | 1 Week | 0.292 |
| Treatment | 2015–2016 | None | 0.264 |
| Key Facts, Prevention, Treat Flu, Vaccine | 2014–2015 | None | 0.248 |
| Key Facts | 2014–2015 | None | 0.247 |
| FluView, Symptoms, Treatment | 2015–2016 | None | 0.232 |
| Antivirals | 2012–2013 | None | 0.225 |
| Key Facts, Prevention, Treat Flu, Vaccine | 2012–2013 | None | 0.223 |
| Symptoms | 2015–2016 | None | 0.220 |
| Antivirals, Symptoms | 2015–2016 | None | 0.220 |
| Key Facts, Prevention, Treat Flu, Vaccine | 2014–2015 | 1 Week | 0.220 |
| Treat Flu | 2012–2013 | None | 0.213 |
| Treatment | 2013–2014 | None | 0.176 |
| Treat Flu | 2015–2016 | None | 0.174 |
| FluView | 2015–2016 | None | 0.173 |
| Prevention | 2012–2013 | None | 0.162 |
| FluView, Symptoms, Treatment | All | None | 0.150 |
| All | 2015–2016 | None | 0.140 |
| Flu Basics | 2015–2016 | None | 0.137 |
| FluView, Symptoms, Treatment | 2013–2014 | None | 0.133 |
| All | 2014–2015 | None | 0.130 |
| FluView, Symptoms, Treatment | 2015–2016 | 1 Week | 0.129 |
| Key Facts, Prevention, Treat Flu, Vaccine | 2013–2014 | 1 Week | 0.116 |
| Antivirals, Symptoms | 2015–2016 | 1 Week | 0.114 |
| FluView, Symptoms, Treatment | 2013–2014 | 1 Week | 0.111 |
| All | All | None | 0.110 |
| High Risk Complications | 2014–2015 | None | 0.102 |
| All | 2014–2015 | 1 Week | 0.102 |
| All | 2014–2015 | 2 Week | 0.102 |
| Key Facts, Prevention, Treat Flu, Vaccine | 2013–2014 | None | 0.098 |
| Treatment | 2012–2013 | None | 0.087 |
| Treat Flu | 2013–2014 | None | 0.082 |
| Treat Flu | 2014–2015 | None | 0.075 |
| Symptoms | 2013–2014 | None | 0.074 |
| Antivirals, Symptoms | 2013–2014 | None | 0.068 |
| Key Facts | 2013–2014 | None | 0.060 |
| Antivirals | 2015–2016 | None | 0.049 |
| Antivirals, Symptoms | 2013–2014 | 1 Week | 0.046 |
| High Risk Complications | 2013–2014 | None | 0.045 |
| All | 2015–2016 | 1 Week | 0.042 |
| Antivirals | 2013–2014 | None | 0.038 |
| Flu Basics | 2013–2014 | None | 0.038 |
| Prevention | 2014–2015 | None | 0.035 |
| Prevention | 2015–2016 | None | 0.022 |
| High Risk Complications | 2015–2016 | None | 0.013 |
| All | 2013–2014 | None | 0.011 |
| All | 2013–2014 | 2 Week | 0.010 |
| Prevention | 2013–2014 | None | 0.002 |
| Flu Basics | 2014–2015 | None | 0.001 |
| All | 2013–2014 | 1 Week | 0.001 |
| All | 2015–2016 | 2 Week | 0.000 |

## *Wisconsin*

| **Pages Used in Model** | **Season(s)** | **Shift** | $\boldsymbol{r}^{\boldsymbol{2}}$ |
| --- | --- | --- | --- |
| All | 2012–2013 | None | 0.801 |
| FluView, Symptoms, Treatment | 2012–2013 | 1 Week | 0.774 |
| Antivirals, Symptoms | 2012–2013 | None | 0.772 |
| Symptoms | 2012–2013 | None | 0.755 |
| All | 2012–2013 | 1 Week | 0.742 |
| Antivirals, Symptoms | 2012–2013 | 1 Week | 0.733 |
| FluView | 2012–2013 | None | 0.722 |
| FluView, Symptoms, Treatment | 2013–2014 | 1 Week | 0.720 |
| All | 2012–2013 | 2 Week | 0.693 |
| Antivirals, Symptoms | 2013–2014 | 1 Week | 0.644 |
| Treatment | 2013–2014 | None | 0.635 |
| FluView | 2014–2015 | None | 0.615 |
| Key Facts, Prevention, Treat Flu, Vaccine | 2013–2014 | 1 Week | 0.613 |
| Prevention | 2014–2015 | None | 0.570 |
| FluView, Symptoms, Treatment | 2013–2014 | None | 0.568 |
| Treatment | 2012–2013 | None | 0.548 |
| Key Facts | 2013–2014 | None | 0.546 |
| Key Facts, Prevention, Treat Flu, Vaccine | 2013–2014 | None | 0.537 |
| All | 2015–2016 | 1 Week | 0.498 |
| FluView, Symptoms, Treatment | 2014–2015 | 1 Week | 0.483 |
| Flu Basics | 2012–2013 | None | 0.477 |
| All | 2015–2016 | 2 Week | 0.475 |
| FluView, Symptoms, Treatment | 2014–2015 | None | 0.464 |
| Antivirals, Symptoms | 2013–2014 | None | 0.454 |
| Flu Basics | 2013–2014 | None | 0.451 |
| Antivirals, Symptoms | 2014–2015 | 1 Week | 0.445 |
| Antivirals, Symptoms | 2014–2015 | None | 0.444 |
| Symptoms | 2014–2015 | None | 0.442 |
| Symptoms | 2013–2014 | None | 0.437 |
| Prevention | 2013–2014 | None | 0.432 |
| FluView | 2013–2014 | None | 0.411 |
| All | 2015–2016 | None | 0.389 |
| Antivirals, Symptoms | 2015–2016 | 1 Week | 0.388 |
| FluView, Symptoms, Treatment | 2015–2016 | 1 Week | 0.381 |
| Treatment | 2014–2015 | None | 0.356 |
| Prevention | 2012–2013 | None | 0.353 |
| Symptoms | 2015–2016 | None | 0.351 |
| Antivirals, Symptoms | 2015–2016 | None | 0.351 |
| FluView, Symptoms, Treatment | 2015–2016 | None | 0.343 |
| Antivirals | 2012–2013 | None | 0.324 |
| All | 2013–2014 | 1 Week | 0.310 |
| Treatment | 2015–2016 | None | 0.298 |
| Key Facts | 2012–2013 | None | 0.296 |
| Vaccine | 2013–2014 | None | 0.264 |
| All | 2014–2015 | 1 Week | 0.250 |
| Vaccine | 2014–2015 | None | 0.240 |
| Key Facts | 2014–2015 | None | 0.235 |
| Treat Flu | 2012–2013 | None | 0.231 |
| All | 2014–2015 | None | 0.230 |
| Flu Basics | 2014–2015 | None | 0.223 |
| FluView, Symptoms, Treatment | All | None | 0.220 |
| Key Facts, Prevention, Treat Flu, Vaccine | 2012–2013 | None | 0.218 |
| Key Facts, Prevention, Treat Flu, Vaccine | 2012–2013 | 1 Week | 0.212 |
| Antivirals | 2014–2015 | None | 0.208 |
| Key Facts, Prevention, Treat Flu, Vaccine | 2014–2015 | 1 Week | 0.206 |
| Key Facts, Prevention, Treat Flu, Vaccine | 2014–2015 | None | 0.197 |
| Flu Basics | 2015–2016 | None | 0.194 |
| Treat Flu | 2013–2014 | None | 0.187 |
| All | 2014–2015 | 2 Week | 0.187 |
| All | 2013–2014 | None | 0.187 |
| All | 2013–2014 | 2 Week | 0.171 |
| Antivirals | 2013–2014 | None | 0.159 |
| Key Facts, Prevention, Treat Flu, Vaccine | 2015–2016 | None | 0.150 |
| All | All | None | 0.148 |
| Key Facts, Prevention, Treat Flu, Vaccine | 2015–2016 | 1 Week | 0.145 |
| Antivirals | 2015–2016 | None | 0.140 |
| Key Facts | 2015–2016 | None | 0.137 |
| Vaccine | 2015–2016 | None | 0.135 |
| High Risk Complications | 2012–2013 | None | 0.071 |
| Prevention | 2015–2016 | None | 0.050 |
| FluView | 2015–2016 | None | 0.043 |
| High Risk Complications | 2014–2015 | None | 0.040 |
| Treat Flu | 2014–2015 | None | 0.033 |
| Vaccine | 2012–2013 | None | 0.028 |
| Treat Flu | 2015–2016 | None | 0.021 |
| High Risk Complications | 2013–2014 | None | 0.019 |
| High Risk Complications | 2015–2016 | None | 0.002 |
